# *Arabidopsis* metacaspase MC1 localizes in stress granules, clears protein aggregates, and delays senescence

**DOI:** 10.1093/plcell/koad172

**Published:** 2023-07-01

**Authors:** Nerea Ruiz-Solaní, Jose Salguero-Linares, Laia Armengot, Jaime Santos, Irantzu Pallarès, Katarina P van Midden, Ujjal J Phukkan, Seda Koyuncu, Júlia Borràs-Bisa, Liang Li, Crina Popa, Frederik Eisele, Anna Maria Eisele-Bürger, Sandra Malgrem Hill, Emilio Gutiérrez-Beltrán, Thomas Nyström, Marc Valls, Ernesto Llamas, David Vilchez, Marina Klemenčič, Salvador Ventura, Nuria S Coll

**Affiliations:** Centre for Research in Agricultural Genomics (CRAG), CSIC-IRTA-UAB-UB, Bellaterra 08193, Spain; Department of Genetics, Microbiology and Statistics, Universitat de Barcelona, Barcelona 08028, Spain; Centre for Research in Agricultural Genomics (CRAG), CSIC-IRTA-UAB-UB, Bellaterra 08193, Spain; Centre for Research in Agricultural Genomics (CRAG), CSIC-IRTA-UAB-UB, Bellaterra 08193, Spain; Department of Genetics, Microbiology and Statistics, Universitat de Barcelona, Barcelona 08028, Spain; Institut de Biotecnologia i de Biomedicina, Departament de Bioquímica i Biologia Molecular, Universitat Autònoma de Barcelona, Barcelona 08193, Spain; Institut de Biotecnologia i de Biomedicina, Departament de Bioquímica i Biologia Molecular, Universitat Autònoma de Barcelona, Barcelona 08193, Spain; Department of Chemistry and Biochemistry, Faculty of Chemistry and Chemical Technology, University of Ljubljana, Ljubljana 1000, Slovenia; Centre for Research in Agricultural Genomics (CRAG), CSIC-IRTA-UAB-UB, Bellaterra 08193, Spain; Cologne Excellence Cluster for Cellular Stress Responses in Aging-Associated Diseases (CECAD), University of Cologne, Cologne 50931, Germany; Centre for Research in Agricultural Genomics (CRAG), CSIC-IRTA-UAB-UB, Bellaterra 08193, Spain; Centre for Research in Agricultural Genomics (CRAG), CSIC-IRTA-UAB-UB, Bellaterra 08193, Spain; Centre for Research in Agricultural Genomics (CRAG), CSIC-IRTA-UAB-UB, Bellaterra 08193, Spain; Department of Microbiology and Immunology, The Sahlgrenska Academy at the University of Gothenburg, Gothenburg 41390, Sweden; Department of Microbiology and Immunology, The Sahlgrenska Academy at the University of Gothenburg, Gothenburg 41390, Sweden; Department of Microbiology and Immunology, The Sahlgrenska Academy at the University of Gothenburg, Gothenburg 41390, Sweden; Instituto de Bioquímica Vegetal y Fotosíntesis (Universidad de Sevilla and Consejo Superior de Investigaciones Científicas), 41092 Seville, Spain; Departamento de Bioquímica Vegetal y Biología Molecular, Facultad de Biología, Universidad de Sevilla, Sevilla 41012, Spain; Department of Microbiology and Immunology, The Sahlgrenska Academy at the University of Gothenburg, Gothenburg 41390, Sweden; Centre for Research in Agricultural Genomics (CRAG), CSIC-IRTA-UAB-UB, Bellaterra 08193, Spain; Department of Genetics, Microbiology and Statistics, Universitat de Barcelona, Barcelona 08028, Spain; Cluster of Excellence on Plant Sciences (CEPLAS), Institute for Plant Sciences, University of Cologne, Cologne D-50674, Germany; Cologne Excellence Cluster for Cellular Stress Responses in Aging-Associated Diseases (CECAD), University of Cologne, Cologne 50931, Germany; Center for Molecular Medicine Cologne (CMMC), University of Cologne, Cologne 50931, Germany; Faculty of Medicine, University Hospital Cologne, Cologne 50931, Germany; Department of Chemistry and Biochemistry, Faculty of Chemistry and Chemical Technology, University of Ljubljana, Ljubljana 1000, Slovenia; Institut de Biotecnologia i de Biomedicina, Departament de Bioquímica i Biologia Molecular, Universitat Autònoma de Barcelona, Barcelona 08193, Spain; Centre for Research in Agricultural Genomics (CRAG), CSIC-IRTA-UAB-UB, Bellaterra 08193, Spain; Consejo Superior de Investigaciones Científicas (CSIC), Barcelona 08001, Spain

## Abstract

Stress granules (SGs) are highly conserved cytoplasmic condensates that assemble in response to stress and contribute to maintaining protein homeostasis. These membraneless organelles are dynamic, disassembling once the stress is no longer present. Persistence of SGs due to mutations or chronic stress has been often related to age-dependent protein-misfolding diseases in animals. Here, we find that the metacaspase MC1 is dynamically recruited into SGs upon proteotoxic stress in *Arabidopsis* (*Arabidopsis thaliana*). Two predicted disordered regions, the prodomain and the 360 loop, mediate MC1 recruitment to and release from SGs. Importantly, we show that MC1 has the capacity to clear toxic protein aggregates in vivo and in vitro, acting as a disaggregase. Finally, we demonstrate that overexpressing MC1 delays senescence and this phenotype is dependent on the presence of the 360 loop and an intact catalytic domain. Together, our data indicate that MC1 regulates senescence through its recruitment into SGs and this function could potentially be linked to its remarkable protein aggregate-clearing activity.

## Teaser

The *Arabidopsis* metacaspase MC1 is recruited to stress granules upon proteotoxic stress, participates in clearance of pathological protein aggregates, and delays senescence.

## Introduction

To cope with stress, eukaryotic cells are equipped with multiple sophisticated mechanisms that ultimately confer robustness against various perturbations. As part of their stress responses, cells must readjust proteostasis (protein homeostasis), which is achieved through an arrest in protein synthesis and activation of protein quality control (PQC) mechanisms to prevent accumulation of misfolded proteins in the cytoplasm, potentially causing proteotoxicity (Alberti and Carra 2018). The proteostatic capacity of cells declines with age, which may reduce their capacity to dispose of potentially harmful protein aggregates (Vilchez et al. 2014; Hipp et al. 2019).

An important stress response mechanism in fungi, animals, and plants is the formation of stress granules (SGs). SGs are biomolecular condensates assembled in the cytosol under stress conditions with a highly dynamic behavior, containing a combination of mRNA and proteins, many of which have RNA-binding ability (Jain et al. 2016; Markmiller et al. 2018; Youn et al. 2019). These membraneless compartments were originally viewed as sites of accumulation and disposal of stalled mRNAs but are currently emerging as major orchestrators of stress responses (Buchan and Parker 2009; Maruri-López et al. 2021).

Current models predict that SG formation is mediated by liquid–liquid phase separation (LLPS) promoted by multivalent molecules, such as proteins featuring low complexity regions (LCRs)/intrinsically disordered regions (IDRs) (Protter and Parker 2016). Assembly and clearance of SGs are finely regulated, with initial formation of a dense core by LLPS followed by recruitment of peripheral proteins (Jain et al. 2016; Markmiller et al. 2018). Core components are proteins containing IDRs and RNA-binding domains together with proteins involved in translation, whereas the shell is composed of an array of mRNA, proteins, and small molecules that vary depending on the species, cell type, and developmental stage (González-García et al. 2011; Jain et al. 2016; Niewidok et al. 2018; Kosmacz et al. 2019; Guillén-Boixet et al. 2020).

Compared to yeast and mammals, little is known about plant SGs, despite their important role in stress responses, including heat, hypoxia, salt, and drought (Sorenson and Bailey-Serres 2014; Yan et al. 2014; Gutierrez-Beltran et al. 2015; Marondedze et al. 2020). Among these stress responses, heat is the best characterized so far, as it presents an archetypal form of acute stress resulting in proteotoxicity that must be handled by various mechanisms, including SG formation (Maruri-López et al. 2021). SGs provide efficient regulatory platforms under stress conditions (Maruri-López et al. 2021), serving as (i) mRNA reorganization centers, wherein their fate is determined (reinitiation, decay, and storage), (ii) temporary protein storage centers, to protect them from unfolding, and (iii) enzyme recruitment centers, to facilitate rapid activation of certain metabolic pathways. Plant SG component catalogs and molecular markers have started to become available in recent years opening new avenues of research.

An essential property of SGs is their dynamism: to be functional, they must be inducible and reversible. In mammals, cumulative evidence links altered SG dynamics with pathologies featuring aberrant protein coalescence leading to aggregation (Baradaran-Heravi et al. 2020; Marcelo et al. 2021). In several neurodegenerative diseases, mutations in LCRs/IDRs of certain proteins disrupt their biophysical properties, leading to enhanced LLPS and formation of pathological protein aggregates (Baradaran-Heravi et al. 2020). Pathological SGs undergo a liquid-to-solid transition and persist even after the stress has passed, acting as undissolvable protein traps. This is the case of polyglutamine (polyQ) pathologies, such as Huntington’s disease, caused by abnormal polyQ extensions, that make them more aggregation prone (Sanchez et al. 2021).

Plants may have evolved extremely efficient mechanisms to deal with toxic protein aggregation. It has been recently demonstrated that overexpression of synthetic extended polyQ proteins, which normally aggregate and cause cell death in animal models, does not cause deleterious defects in plants (Llamas et al. 2022). In fact, plants overexpressing synthetic protein variants that constitutively aggregate do not show major defects (Jung et al. 2020; Llamas et al. 2022). This may indicate that plants have evolved extremely efficient mechanisms to deal with protein aggregation. Selective autophagy has been previously implicated in degradation of protein aggregates or aggrephagy during proteotoxic stress (Jung et al. 2020). However, whether formation of these protein aggregates is related to molecular condensation including SG formation and dynamics has not been addressed.

Metacaspases are cysteine proteases present in plants, yeast, and protozoa (Uren et al. 2000). Plant metacaspases are divided into Type I if they bear an N-terminal prodomain and Type II, if no prodomain is present but instead a long linker between the catalytic subunits exists (Uren et al. 2000; Klemenčič and Funk 2019). Several metacaspases have been shown to play important roles in stress responses (Coll et al. 2010; Hander et al. 2019; Minina et al. 2020; Luan et al. 2021; Pitsili et al. 2023), although in most cases the mode of action of these proteases remains obscure. The model plant *Arabidopsis thaliana* (hereafter *Arabidopsis*) encodes 9 metacaspases in its genome. MC1-3 (AtMC1-3/AtMCA-Ia-c) is a Type I metacaspase, while MC4-9 (AtMC4-9/AtMCA-IIa-f) is a Type II metacaspase (Minina et al. 2020). We previously showed that plants lacking MC1 exhibit accelerated senescence and accumulate aggregated proteins, indicating a potential role of MC1 in proteostasis (Coll et al. 2014). In addition, a portion of MC1 relocalizes to insoluble protein deposits (IPODs) under proteotoxic stress conditions. Data from our lab and others indicate that MC1 may help stabilizing various proteins (Roberts et al. 2013; Lema Asqui et al. 2018; Wang et al. 2021). However, the specific mechanisms whereby MC1 contributes to protein stabilization and aggregate clearance remain unknown.

Here, we demonstrate that MC1 is dynamically recruited to SGs upon proteotoxic stress. This SG localization is mediated by a C-terminal IDR, the 360 loop. We show that MC1 participates in the clearance of pathological aggregates in evolutionarily distant organisms ranging from yeast and animals to plants. In vitro, recombinant MC1 (rMC1) alone acts as a highly efficient disaggregase. In plants, this function can be harnessed to delay senescence, as observed in MC1 overexpressing lines.

## Results

### MC1 dynamically localizes to cytoplasmic SGs upon acute proteotoxic stress

**Figure 1.**
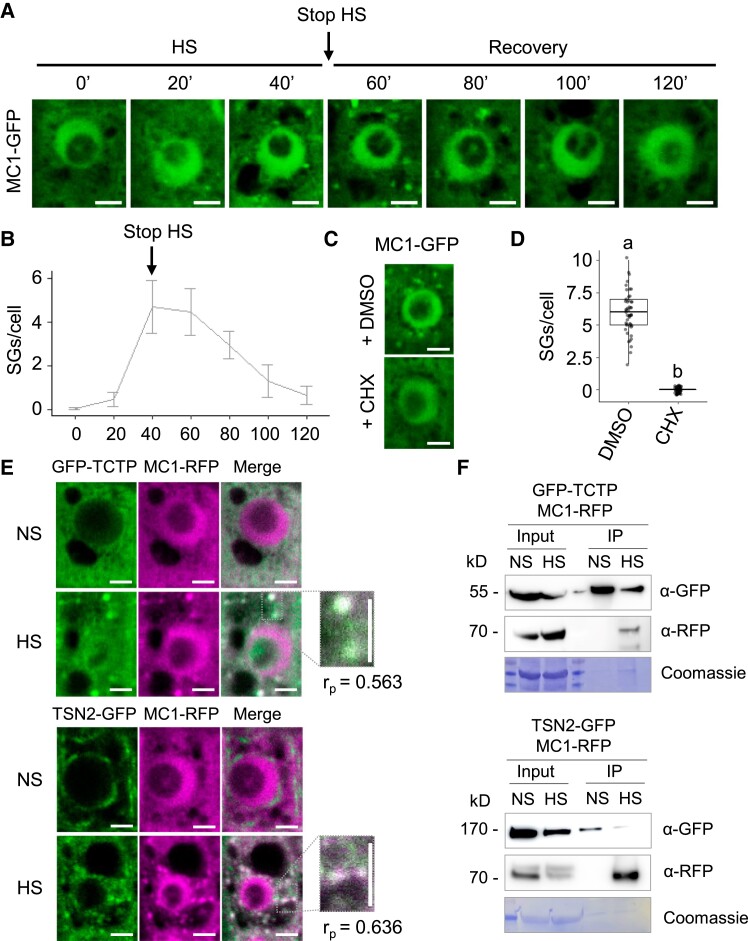
MC1 is recruited to SGs upon heat stress. **A)** Five-day-old *mc1 Arabidopsis* seedlings expressing *Pro35S:MC1-GFP* were heat stressed at 39 °C for 40 min (HS), followed by incubation at 22 °C for up to 120 min (recovery). Images of root tips were taken at indicated time points. Bars = 5 *μ*m. **B)** Kinetics of the assembly and disassembly of MC1 cytoplasmic foci. Graph shows means ± Sd of 3 independent experiments, each including 5 seedlings. Eight to 10 cells for each seedling were analyzed for SG quantification. “Stop HS” corresponds to the time point when plants were transferred from 39 °C to 22 °C. **C)** Treatment with CHX inhibits the formation of MC1 foci in root tip cells. For CHX treatment, 5-d-old seedlings expressing *Pro35S:MC1-GFP* were incubated with 200 ng/*μ*l CHX for 30 min at 22 °C before HS. Images show localization of MC1-GFP in heat-stressed (39 °C for 40 min) root tip cells of 5-d-old seedlings previously treated with CHX or DMSO (control). **D)** Quantification of MC1-GFP foci in the experiment shown in **C)**. Upper and lower box boundaries represent the first and third quantiles, respectively; horizontal lines mark the median and whiskers mark the highest and lowest values. Three independent experiments, each containing at least 5 individual measurements, were performed. Means with different letters are significantly different at *P* < 0.05 (1-way ANOVA). **E)** Colocalization of GFP-TCTP or TSN2-GFP with MC1-RFP in heat-stressed (39 °C for 40 min) root tip cells of 5-d-old seedlings expressing both *Pro35S:GFP-TCTP* or *Pro35S:TSN2-GFP* together with *Pro35S:MC1-RFP*. Inset show enlarged boxed areas. Pearson coefficients (*r_P_*) of colocalization of GFP-TCTP, TSN2-GFP, and MC1-RFP represent the mean of 5 replicate measurements from 3 independent experiments. Scale bars = 5 *μ*m. **F)** Immunoprecipitation (IP) of GFP-TCTP or TSN2-GFP and MC1-RFP in protein extracts prepared from leaves of 3-wk-old transgenic *Arabidopsis* seedlings expressing either *Pro35S:GFP-TCTP* or *Pro35S:TSN2-GFP* with *Pro35S:MC1-RFP*. Samples were kept in control (NS) conditions or heat stressed (HS, 39 °C for 40 min). Input and IP fractions were analyzed by immunoblotting using α-GFP or α-RFP.

To gain a deeper understanding of MC1 function, we generated transgenic lines expressing *MC1* tagged with a GFP under the control of the *35S* promoter in the *mc1* mutant background (*mc1 Pro35S:MC1-GFP*) and evaluated its subcellular localization. Under basal conditions, MC1-GFP showed a diffuse pattern in both cytoplasm and nucleus (Fig. 1A). Heat stress (HS) treatment (39 °C for 40 min; Gutierrez-Beltran et al. 2015) resulted in rapid formation of dynamic cytoplasmic puncta that grew in size over time and disappeared shortly after returning the plants to nonstress conditions (Figs. 1, A and B, and S1). The same heat-responsive relocalization pattern was observed when *MC1-GFP* was expressed under the control of its native promoter (Supplemental Fig. S2, A and B). To evaluate if such puncta correspond to SGs, we used cycloheximide (CHX), an inhibitor of translational elongation that prevents SG assembly and forces the disassembly of existing SGs (Weber et al. 2008). Application of CHX blocked the appearance of the observed heat stress-induced puncta (Fig. 1, C and D), indicating that they may indeed correspond to SGs.

To further examine if MC1 colocalized with SGs, we used the plant SG markers translationally controlled tumor protein (TCTP) and Tudor staphylococcal nuclease 2 (TSN2), which locate into SGs specifically under HS conditions (Gutierrez-Beltran et al. 2021). We observed that transgenic plants stably coexpressing *MC1-RFP* (*Pro35S:MC1-RFP*) and *GFP-TCTP* (*Pro35S:GFP-TCTP*) or *TSN2-GFP* (*Pro35S:TSN2-GFP*) show cytoplasmic colocalization in SGs under heat stress conditions (Fig. 1E). Furthermore, MC1 immunoprecipitated with both TCTP and TSN2 in transgenic plants subjected to HS (Fig. 1F). Similarly, MC1 colocalized with other well-known SG markers RBP47 and TSN2 (Lorković et al. 2000; Gutierrez-Beltran et al. 2021) in protoplasts from transgenic *MC1-GFP* lines transiently expressing *RFP-RBP47* or *RFP-TSN2* (Supplemental Fig. S3, A and B). Together, these data demonstrate that MC1 dynamically relocalizes to SGs upon heat treatment, disappearing upon stress removal.

### The IDRs of MC1 are aggregation prone and confer insolubility in vitro

**Figure 2.**
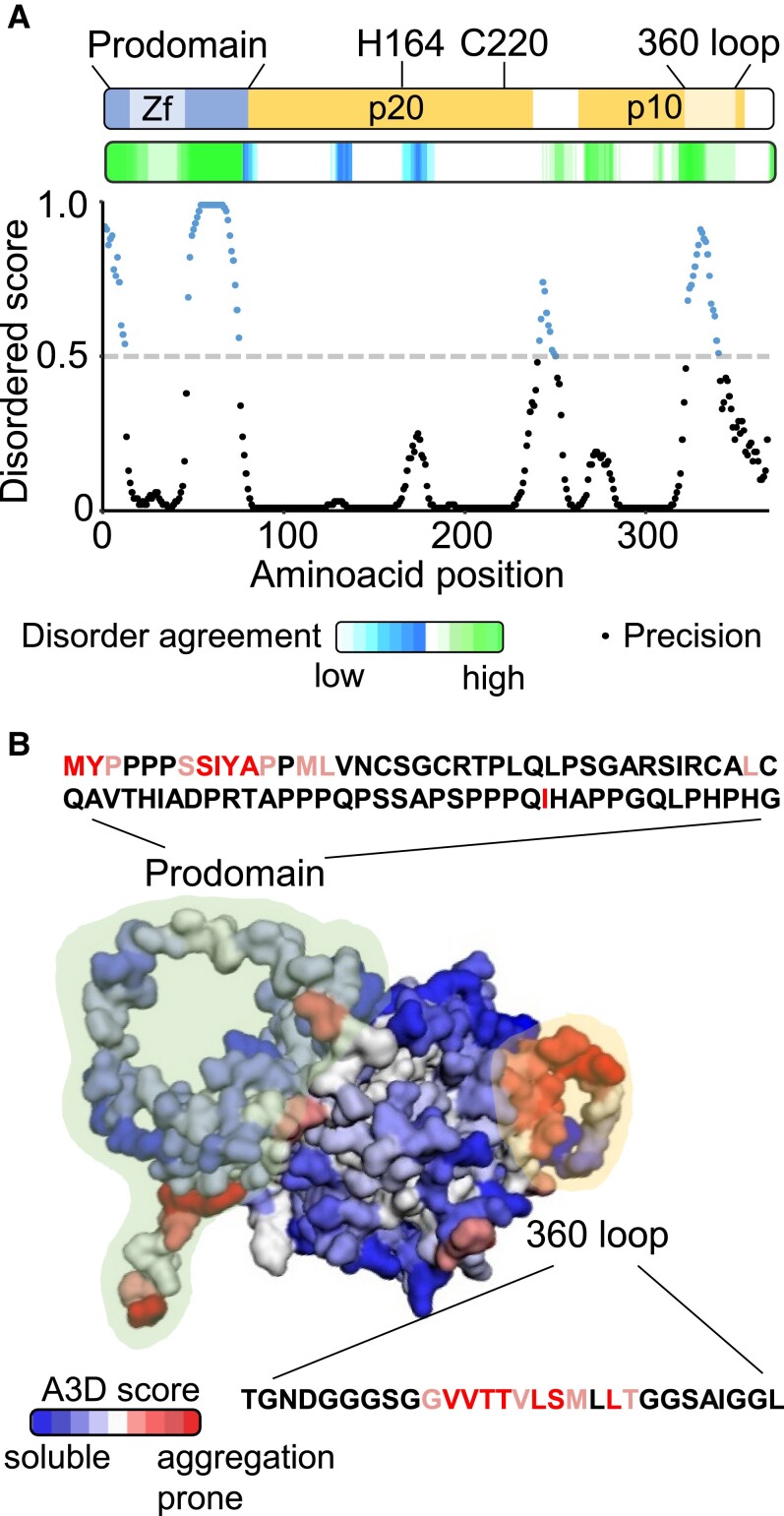
MC1 contains intrinsically disordered and aggregation-prone regions. **A)** Prediction of IDRs of MC1. Top, scheme of MC1 protein structure. Zf: LSD1-Zinc finger domain within the prodomain (amino acids 1-77); H164 and C220 correspond to the amino acids of the catalytic dyad within the large p20 catalytic subunit; 360 loop (amino acids 318-346): hydrophobic loop within the small p10 catalytic subunit. Middle and bottom, prediction of the disordered regions by D^2^P^2^ (https://d2p2.pro) and DISOPRED3 (http://bioinf.cs.ucl.ac.UK/psipred), respectively. Precision above the dotted line show disordered scores higher than 0.5 and precision dots below the dotted line show disordered scores lower than 0.5. B) A3D structure of MC1. The prodomain and the 360 loop are highlighted. The amino acid sequences of the prodomain and 360 loop are shown and the amino acids with high A3D scores (aggregation-prone) are highlighted.

SGs are enriched in proteins containing predicted IDRs (Guillén-Boixet et al. 2020; Gutierrez-Beltran et al. 2021; Schmit et al. 2021). IDRs have been proposed to act as one of the main driving forces of condensate assembly, although the exact mechanism by which this occurs remains to be fully elucidated (Posey et al. 2018; Alberti et al. 2019). We used a combination of 2 predictive algorithms (D^2^P^2^, https://d2p2.pro, and DISOPRED3, http://bioinf.cs.ucl.uk/psipred; Oates et al. 2013; Jones and Cozzetto 2015) to pinpoint potential IDRs within MC1 amino acid sequence. Based on these predictions, MC1 encompasses 2 major IDRs (Fig. 2A), 1 at the N-terminal prodomain and another at a region of the predicted C-terminal p10 catalytic domain known as the 360 loop (van Midden et al. 2021). Since aggregation propensity is also considered an intrinsic determinant of phase separation (Babinchak and Surewicz 2020), we used AGGRESCAN3D (A3D) to predict the structural aggregation propensity of MC1 on top of its AlphaFold predicted structure (http://biocomp.chem.uw.edu.pl/A3D/; Zambrano et al. 2015). Interestingly, MC1 displays a strong propensity to aggregate at the predicted IDRs (Fig. 2B). In particular, the 360 loop shows the longest stretch of amino acids with high aggregation propensity scores.

**Figure 3.**
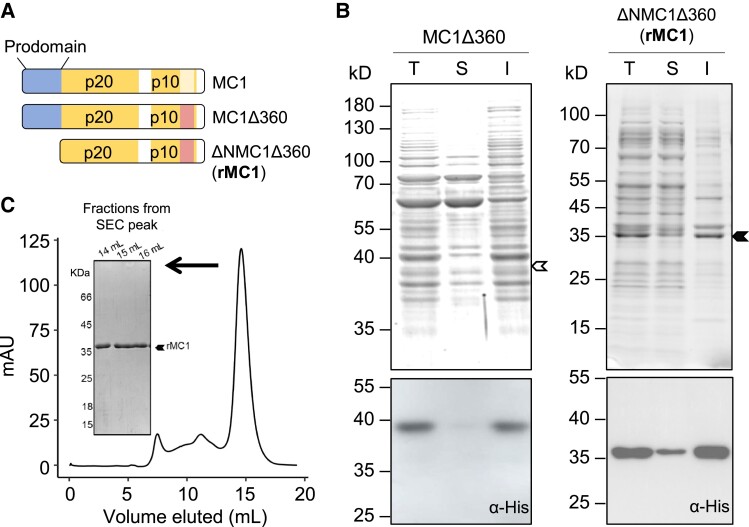
The prodomain and the 360 loop confer insolubility to MC1. **A)** Schematic representation of MC1 full length, MC1Δ360 (without 360 loop) and ΔNMC1Δ360 (without prodomain and 360 loop) domain architecture. **B)** SDS–PAGE Coomassie-stained gels (upper panels) and western blot analysis (lower panels) of lysates from total, soluble, or insoluble fractions of *E. coli* cells expressing either MC1Δ360 or ΔNMC1Δ360 (rMC1) carrying an N-terminal 6xHis tag. Arrow denotes expected molecular weight of each of the 2 MC1 variants. **C)** Size-exclusion chromatography of concentrated eluates obtained by nickel-affinity chromatography (Supplemental Fig. S3A). The inlet shows an SDS–PAGE Coomassie-stained gel of fractions 14 to 16 ml of the eluted volume from a Superdex 75 column.

The 360 loop of MC1 is a highly hydrophobic sequence only present in plant Type I metacaspases (van Midden et al. 2021). In fungi, protozoa, and red algae, Type I metacaspases do not contain the 360 loop and, interestingly, these proteins are soluble when full length is produced recombinantly in vitro (McLuskey et al. 2012; Wong et al. 2012). In contrast, previous efforts to produce recombinant plant Type I metacaspases proved unsuccessful due to the fact that their full-length versions are highly insoluble (van Midden et al. 2021). Removal of the 360 loop and the prodomain was necessary to express soluble MC1 in *Escherichia coli* (Fig. 3, A and B), similar to what was previously shown with the single Type I metacaspase of the green algae *Chlamydomonas reinhardtii* CrMCA-I (van Midden et al. 2021). Removal of the 360 loop alone was not sufficient to solubilize MC1 (Fig. 3B). The soluble MC1 variant devoid of the prodomain and the 360 loop carrying an N-terminal hexahistidine (6xHis) tag (referred to as rMC1) was purified to homogeneity by nickel-affinity chromatography (Supplemental Fig. S4A) and further isolated by size-exclusion chromatography removing minor impurities (Fig. 3C). Importantly, rMC1 was catalytically active as shown by its ability to cleave *Arabidopsis* SERPIN1, an inhibitor and previously reported in planta substrate of MC1 (Lema Asqui et al. 2018) (Supplemental Fig. S5A). rMC1 behaved as a canonical Type I metacaspase, showing dependency on calcium ions at low millimolar concentrations (1 to 10 mM) and a neutral pH (pH 7) for maximum cleavage of the fluorogenic substrate Z-FR-7-amino-4-methylcoumarin (AMC) (Supplemental Fig. S5, B and C, respectively), similar to CrMCA-I (van Midden et al. 2021). In agreement with the observed trypsin-like activity of metacaspases, rMC1 cleaved the trypsin substrate β-casein (rβ-casein) (Supplemental Fig. S5D) (Lee et al. 2007). We also purified rMC1 carrying a point mutation in the catalytic cysteine (C220) to alanine (rMC1CA) (Supplemental Fig. S4, B and C). Importantly, rMC1CA was unable to cleave SERPIN1 or rβ-casein (Supplemental Fig. S5, A and D). Together, these data show that MC1 contains 2 distinct IDRs that are aggregation prone and confer high insolubility for protein overexpression and isolation in vitro. When removed, proteolytically active rMC1 can be expressed and isolated.

### The IDRs of MC1 regulate its dynamic recruitment into SGs

**Figure 4.**
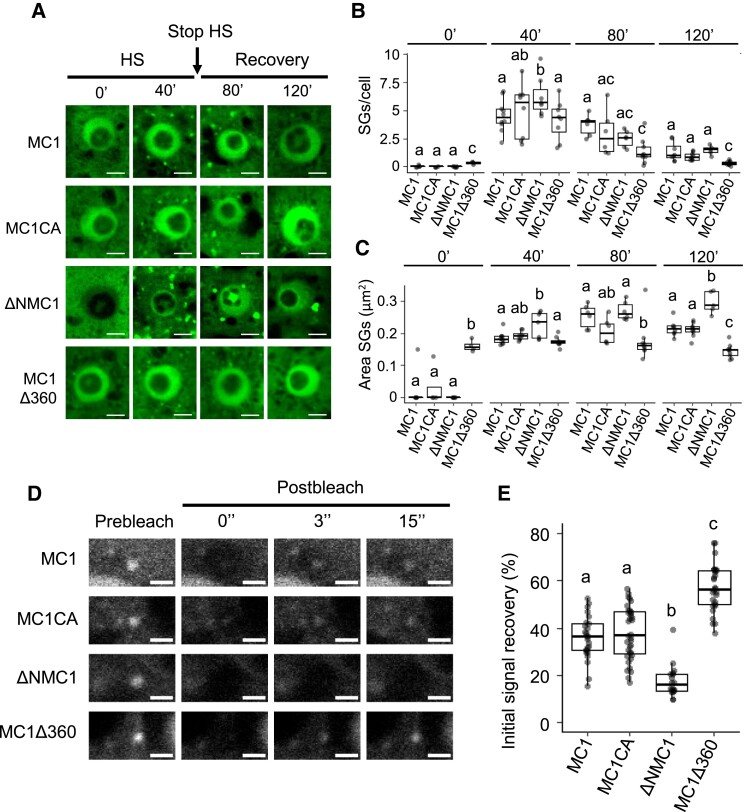
The 360 loop and the prodomain of MC1 are involved, respectively, in recruitment to and clearance from SGs. **A)** Five-day-old *mc1* seedlings expressing *Pro35S:MC1-GFP*, *Pro35S:MC1C220A-GFP*, *Pro35S:ΔNMC1-GFP*, or *Pro35S:MC1Δ360loop-GFP* were heat stressed at 39 °C for 40 min (HS), followed by incubation at 22 °C for up to 120 min (recovery). Images of root tips were taken at indicated time points. Bars = 5 *μ*m. **B)** Quantification of the number of condensates in the experiment shown in **A)**. **C)** Quantification of the area (*µ*m^2^) of condensates in the experiment shown in **B)**. **D)** Selected time frames (prebleach and 0, 3, and 15 s after bleaching) from FRAP analysis of MC1-GFP, MC1C220A-GFP, ΔNMC1-GFP, and MC1Δ360loop-GFP foci formed upon heat stress (40 min at 39 °C) in root tip cells of *mc1* seedlings expressing *Pro35S:MC1-GFP*, *Pro35S:MC1C220A-GFP*, *Pro35S:ΔNMC1-GFP*, or *Pro35S:MC1Δ360loop-GFP*, respectively. Bars = 2 *μ*m. **E)** Initial signal recovery (%) of the experiment shown in **C)**. In **B)**, **C)**, and **E)**, upper and lower box boundaries represent the first and third quantiles, respectively; horizontal lines mark the median and whiskers mark the highest and lowest values. In **B)** and **C)**, 3 independent experiments, each including 3 to 5 seedlings. For each seedling, ∼30 cells from the root meristem were analyzed. In **E)**, 3 independent experiments, each containing at least 8 measurements of different SGs, were performed. Means with different letters are significantly different at *P* < 0.05 (1-way ANOVA).

To determine whether the prodomain and 360 loop IDRs of MC1 are important for its translocation into SGs, we generated transgenic plants stably expressing GFP-tagged truncated versions lacking the prodomain (ΔNMC1) or the 360 loop (MC1Δ360) under the control of the *35S* promoter in the *mc1* mutant background (*mc1 Pro35S:ΔNMC1-GFP* and *mc1 Pro35S:MC1Δ360-GFP*) (Supplemental Fig. S6). We also included transgenic plants carrying a full-length version of MC1 with the catalytic cysteine in position 220 mutated to an alanine (Supplemental Fig. S6), which renders the protease inactive (*mc1 ProMC1:MC1CA-GFP*) (Supplemental Fig. S5, A and D) (Coll et al. 2010; Lema Asqui et al. 2018) to determine whether the catalytic activity of MC1 was required for its SG targeting. Under basal conditions, all MC1 versions showed a diffused cytoplasmic localization (Fig. 4A). Except for ΔNMC1, all other MC1 variants also localized in the nucleus (Fig. 4A). As shown above (Fig. 1), heat stress (39 °C for 40 min) resulted in the rapid recruitment of MC1 into cytoplasmic SGs. Removal of the prodomain resulted in the formation of significantly larger SGs without altering their number per cell, while removal of the 360 loop resulted in smaller and less abundant SGs (Fig. 4, A to C). The catalytically inactive version of MC1 displayed wild-type (WT)–like SGs in response to heat stress (Fig. 4, A to C). Together, these data indicate that the 360 loop may be important for the correct recruitment of MC1 into SGs during stress, while the prodomain may help with the clearance of MC1-containing SGs.

To address the association dynamics of MC1 with SGs, we used fluorescence recovery after photobleaching (FRAP) analysis. MC1-GFP fluorescence partially recovered after photobleaching and mutating the catalytic site of MC1 (*MC1CA-GFP*) did not alter its recovery rate (Fig. 4, C and D). However, mutating the IDRs of MC1 significantly altered the recovery capacity of the protein. On one hand, the few MC1Δ360-GFP-containing SGs showed a higher recovery rate, compared to WT, which suggests that the 360 loop is important for the stable association of MC1 with SGs. (Fig. 4, C and D). In contrast, ΔNMC1-GFP-containing SGs showed slower recovery rate, indicating a potential role of this IDR in the dynamic association of MC1 with SGs. Altogether, these results show that MC1 dynamically associates with SGs. The 360 loop of MC1 might mediate its stable recruitment into SGs while the prodomain might be necessary for its disassembly from SGs.

To further address the role of MC1 in SGs, we measured the dynamics of Rbp47 in WT and MC1-deficient plants. Five-day-old seedlings of WT and *mc1* expressing RFP-Rbp47 were exposed to heat stress (39 °C for 40 min), and the association of Rbp47 with SGs was assessed by FRAP. MC1 deficiency resulted in a reduced exchange rate of RFP-Rbp47 between SGs and the cytoplasm (Supplemental Fig. S7, A and B). This result suggests that MC1 might modulate the association of other SG proteins to SGs.

### MC1 can specifically degrade aggregated proteins

Sustained stress or certain pathological conditions lead to the formation of protein associations or aggregates that, in contrast to SGs, are nonregulated and nondynamic, having detrimental consequences for the cell, tissue, and even at the organismal level (Morimoto 2008). Indeed, SGs may have an important role in the pathogenesis of proteotoxicity-derived conditions, although their exact function remains to be elucidated (Marcelo et al. 2021). Because MC1 is recruited to SGs and we previously observed that *mc1* knock-out mutant plants have increased accumulation of aggregated proteins (Coll et al. 2014), we sought to understand whether its function may be linked to clearance of protein aggregates under stressful/pathological conditions.

**Figure 5.**
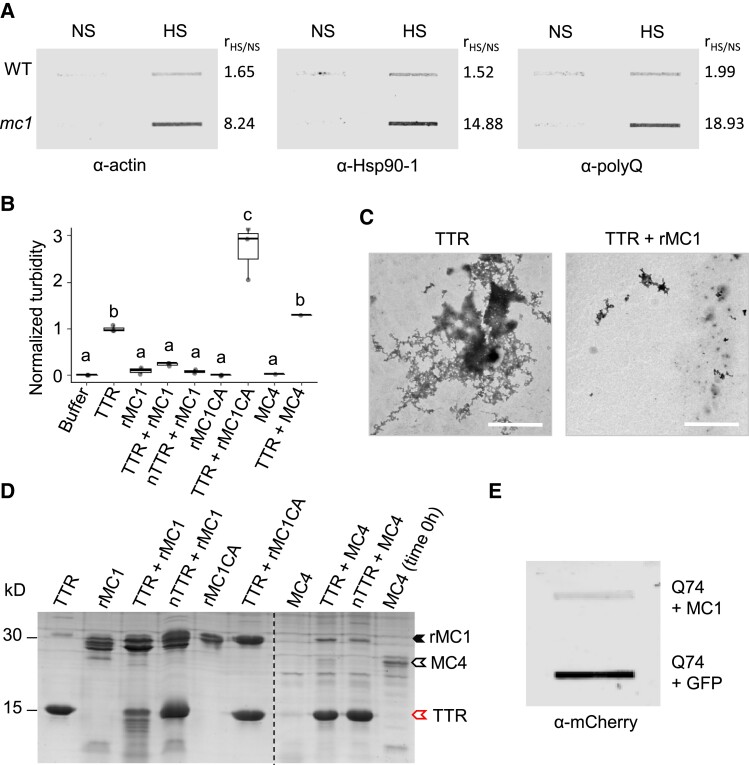
MC1 can specifically clear protein aggregates in vitro and in vivo and the lack of MC1 results in protein aggregate accumulation. **A)** Filter trap analysis of protein extracts from 5-d-old *Arabidopsis mc1* or WT seedlings in control conditions (NS) or subjected to a severe heat stress (HS, 90 min at 37 °C, 90 min at 22 °C and 90 min at 45 °C). SDS-resistant aggregates were detected using antibodies against actin, HSP90-1 or polyQ proteins. r_HS/NS_ represents the ratio between HS and NS protein levels. Signal intensity of the bands was quantified using ImageJ. Two independent experiments were performed with similar results. **B)** Turbidity assays of end-point disaggregation reactions using light scattering at 360 nm. rMC1 and the catalytic inactive form rMC1C220A (rMC1CA) were coincubated with TTR aggregates or native tetrameric TTR for 24 h at 37 °C. rMC1 proteins and TTR aggregates, incubated for the same period, were also measured as controls. Data represent 3 individual measurements. Upper and lower box boundaries represent the first and third quantiles, respectively; horizontal lines mark the median and whiskers mark the highest and lowest values. Means with different letters are significantly different at *P* < 0.05 (1-way ANOVA). **C)** Electron microscopy images of end-point disaggregation reactions of TTR aggregates incubated with or without purified rMC1 for 24 h at 37 °C. **D)** SDS–PAGE analysis of the end-point samples shown in panel **C)**. **E)** Filter trap analysis showing mRFP-Q74 aggregation levels in HEK293 cells. HEK293 cells were transfected with mRFP-Q74 and GFP-MC1 or mRFP-Q74 and GFP as a control. mCherry antibody was used to detect Q74 SDS-resistant aggregates.

First, we investigated whether *Arabidopsis* plants lacking MC1 show defects in protein aggregate clearance and survival after proteotoxic stress. To monitor changes in protein aggregation, we used filter trap analysis, a robust method to detect and quantify protein aggregates, in 5-d-old WT and *mc1* seedlings after heat stress. Seedlings were subjected to 90′ of a moderate HS at 37 °C, followed by 90′ of recovery at 22 °C and a severe HS at 45 °C for 90′. Samples were collected after 1 d of recovery at 22 °C, using nonstressed seedlings as control. We analyzed accumulation of aggregated forms of actin, Hsp90, and proteins containing polyQ stretches, all of them had been shown to aggregate in *Arabidopsis* after heat stress (Llamas et al. 2022). Under basal conditions, protein aggregates (actin, Hsp90-tagged, or polyQ-containing proteins) are barely detectable both in WT and *mc1* mutants, as they can be efficiently cleared by PQC mechanisms (Figs. 5A and S8, A to D). Stresses such as HS result in a sudden overaccumulation of misfolded proteins that often surpasses the PQC capacity of the cell and results in protein aggregation detectable by filter trap (Fig. 5A). Plants lacking MC1 accumulated higher quantities of aggregated proteins than WT after proteotoxic stress, indicating a reduced capacity to manage protein aggregation and proteotoxic stress in the mutant. The overaccumulation of protein aggregates in *mc1* mutants did not affect their thermotolerance (Supplemental Fig. S8A), possibly owing to the fact that the experiment was performed on very young plants to avoid age as an additive effect and these may be extremely proficient at dealing with protein aggregation.

Second, we assessed the capacity of MC1 to disassemble pathological protein aggregates in vitro. We coincubated equimolar concentrations of rMC1 with aggregates of human transthyretin (TTR). TTR is a homotetrameric thyroxine transport protein in which tetramer dissociation events lead to aggregation (Westermark et al. 1990; Quintas et al. 2001). Extracellular insoluble deposits of TTR in several human organs give rise to distinct progressive and fatal clinical syndromes known as TTR amyloidosis (Goren et al. 1980; Hou et al. 2007; Rapezzi et al. 2010). Using turbidity measurements to monitor protein aggregation, we observed that rMC1 treatment caused a 90% reduction of TTR aggregates (Fig. 5B). This protein aggregate clearance activity was dependent on MC1 catalytic activity, as evidenced by the absence of disaggregation in the catalytically dead mutant rMC1CA. Indeed, a 3-fold higher turbidity signal was observed in the rMC1CA-treated samples suggesting that inactive MC1 becomes aggregated when TTR insoluble assemblies are present in the reaction. Visual inspection of TTR samples by transmission electron microscopy (TEM) (Fig. 5C) confirmed the disaggregation activity of MC1. The need of catalytic activity for disaggregation is consistent with the observation that aggregated TTR becomes significantly degraded in presence of rMC1 (Lane 3), as demonstrated by SDS–PAGE (Fig. 5D). Notably, rMC1 acts as a specific disaggregase, clearing protein aggregates but not the functional form of proteins, since it is unable to degrade soluble TTR in its native tetrameric state (nTTR) (Fig. 5D, Lane 3 and 4, respectively). The obtained data indicate that rMC1 targets and disassembles specifically the aggregated and pathogenic form of TTR. Notably, this disaggregase activity toward TTR aggregates was not observed in samples incubated with MC4 (Fig. 5, B and D). We confirmed the activity of the protease by its rapid autoprocessing in the presence of calcium (Fig. 5D, Lane 10), as previously described (Zhu et al. 2020).

Finally, we tested the capacity of MC1 to degrade protein aggregates in vivo. To this end, we used 2 well-established model systems: (i) human embryonic kidney (HEK) cells expressing a polyQ-expanded Huntingtin form (Q74) that causes aggregation and proteotoxicity used as a proxy for the neurodegenerative Huntington's disease (Jimenez-Sanchez et al. 2015) and (ii) yeast expressing a constitutively misfolded carboxypeptidase (ΔssCPY∗) that forms insoluble protein aggregates upon stress (Park et al. 2007). Coexpression of full-length MC1 with Q74 fused to mCherry in HEK cells resulted in a reduction of protein aggregates in comparison to expression of Q74 fused to mCherry alone, as demonstrated by filter trap analysis using anti-mCherry antibody (Figs. 5E and S6E). In yeast, we expressed ΔssCPY∗ fused to the prototrophic marker Leu2 and a C-terminal myc tag (ΔssCL∗) in WT, a mutant lacking the single metacaspase gene *MCA1* in yeast (*ymca1Δ*) and *ymca1Δ* complemented with a WT copy of the *Arabidopsis* MC1. All strains grew normally on control media (Supplemental Fig. S6A, left panel), while on selective media, lacking leucine WT yeast had reduced growth capacity due to degradation of misfolded ΔssCL∗ by PQC systems (Supplemental Figs. S7A, right panel, and S7B). In contrast and as previously shown, *ymca1Δ* was not able to degrade ΔssCL∗ and therefore could grow normally on a leucine-selective media (Hill et al. 2014). This phenotype could be fully complemented by AtMC1 that due to its ability to degrade, ΔssCL∗ restored yeast WT growth levels (Supplemental Figs. S7A, right panel, and S7B). Altogether, these data demonstrate that MC1 can degrade protein aggregates in vitro and in vivo and a lack of MC1 leads to abnormal protein aggregate accumulation under proteotoxic stress.

### MC1 delays senescence

**Figure 6.**
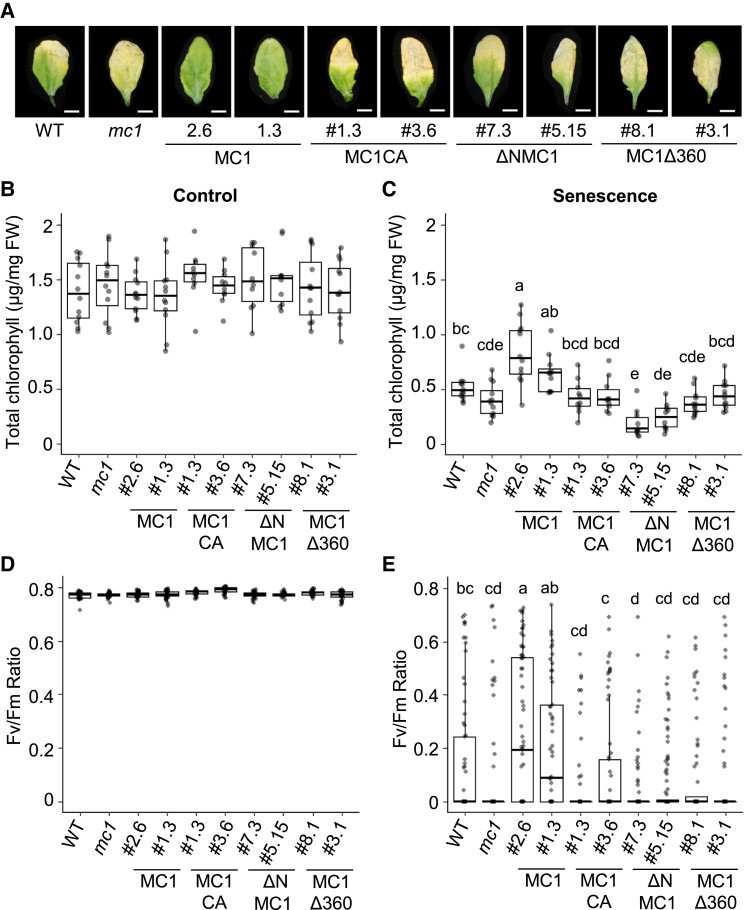
Overexpression of MC1 delays dark-induced senescence. **A)** Representative leaf images of *Arabidopsis* WT, *mc1* mutants and *Pro35S:MC1-GFP mc1*, *ProMC1:MC1C220A-GFP mc1*, *Pro35S:ΔNMC1-GFP mc1*, and *Pro35S:MC1Δ360loop-GFP mc1* grown for 3 wk under controlled growth conditions (16-h light/8-h dark photoperiod) and covered for 8 d to induce senescence. For each plant, only Leaves 5 and 6 were either dark acclimated or used as controls. Bars = 0.5 cm. **B)** and **C)** Total chlorophyll concentration (*µ*g/mg FW) of uncovered (control, **B**) or covered (senescence, **C**) leaves of 3-wk-old *Arabidopsis* WT, *mc1* mutants, and *Pro35S:MC1-GFP mc1*, *ProMC1:MC1C220A-GFP mc1*, *Pro35S:ΔNMC1-GFP mc1*, and *Pro35S:MC1Δ360loop-GFP mc1.* Means with different letters are significantly different at *P* < 0.05 (1-way ANOVA). **D)** and **E)** PSII maximum efficiency (Fv/Fm) quantifications capacity of uncovered (control, **D**) or covered (senescence, **E**) leaves of 3-wk-old *Arabidopsis* WT, *mc1* mutants, and *Pro35S:MC1-GFP mc1*, *ProMC1:MC1C220A-GFP mc1*, *Pro35S:ΔNMC1-GFP mc1*, and *Pro35S:MC1Δ360loop-GFP mc1.* Means with different letters are significantly different at *P* < 0.05 (Kruskal–Wallis test). In **B** to **D)** and **E)**, upper and lower box boundaries represent the first and third quantiles, respectively; horizontal lines mark the median and whiskers mark the highest and lowest values. Four independent experiments, each containing at least 10 leaves for each phenotype, were performed.

Based on all the evidence presented above demonstrating the recruitment of MC1 into SGs and its aggregate clearing function, we hypothesized that overproduction of the protein in plants may minimize the effects of proteotoxic stress occurring during plant aging and contribute to fitness. Previously, we reported that the lack of MC1 led to early senescence in *Arabidopsis* (Coll et al. 2014). Here, we confirmed these results using a dark-induced senescence assay and tested the effects of stably overexpressing WT full-length MC1 and its mutant variants (*MC1*, *MC1CA*, *ΔNMC1*, and *MC1Δ360*) in a *mc1* mutant background. Individual leaves from 3-wk-old plants were covered with aluminum foil, and 8 d later, they were uncovered to evaluate senescence visually and by means of chlorophyll quantification and photosynthetic efficiency. In uncovered leaves (basal conditions), all lines showed similar total chlorophyll levels and photosynthetic efficiency (Fig. 6, B and D). Leaf senescence resulted in a drop in chlorophyll levels and photosynthetic activity in WT plants. In contrast, plants overexpressing *MC1* displayed a clear delay in senescence (Fig. 6A), accompanied by higher chlorophyll levels (Fig. 6C) and higher photosynthetic activity than WT (Fig. 6E).

Importantly, the protease catalytic activity of MC1, the prodomain, and the 360 loop were all required for the observed antiaging phenotype, since *mc1* plants stably expressing MC1CA, ΔNMC1 and MC1Δ360 behaved like WT (Fig. 6, A to E). Reverse transcription quantitative PCR (RT-qPCR) analysis using the senescence marker gene *STAY GREEN1* (*SGR1*; Sakuraba et al. 2012) confirmed the delayed senescence phenotype in plants overexpressing MC1 (Supplemental Fig. S10). Importantly, dark-induced senescence resulted in the formation of cytoplasmic condensates containing the SG marker TSN2 and MC1 (Supplemental Fig. S11). This may indicate that SGs may also form during developmental processes leading to proteotoxicity such as senescence, although further research will be needed to determine the nature and dynamics of these compartments.

## Discussion

SGs are membraneless organelles formed by LLPS under stress conditions and act both as storage compartments and microreactors where signaling takes place (Alberti and Carra 2018). Their functionality inside healthy cells is linked to their ability to assemble and disassemble dynamically in response to changing environments (i.e. assembling upon stress perception and disassembling when the causative stress subdues). SGs contain a high proportion of proteins bearing IDRs/LCRs, which, together with RNA, drive formation of the condensate but at the same time are extremely misfolding/aggregation prone. Therefore, they are closely surveilled by the PQC machinery. Reduced proteostatic capacity derived from aging and mutations affecting the phase separation behavior of these proteins lead to chronic activation of integrated stress responses. This will eventually surpass the PQC capacity of the cell, resulting in the accumulation of misfolded and aggregated proteins and leading to the formation of persistent SGs, which are linked to disease (Wolozin 2012; Wang et al. 2022).

Sessile organisms such as plants cannot flee from extreme and prolonged stress situations, such as heat or drought. Therefore, they must be equipped with extremely efficient PQC mechanisms to deal with massive protein misfolding and aggregation. In fact, proteins containing aggregation-prone polyQ proteins are enriched in plants, but no polyQ pathologies have been reported, in contrast to animals (Llamas et al. 2022). In this context, it has been recently shown that the chloroplasts could act as important protein degradation machines to maintain proteostasis of polyQ-containing proteins (Llamas et al. 2022). Plants may also respond to proteotoxic stress by actively regulating protein solubility and phase behavior, similar to yeast that can tolerate high levels of insoluble proteins and form solid-like condensates (Franzmann and Alberti 2019). Insoluble proteins within these condensates, including SGs, may have evolved to become stress sensors serving an adaptive function (Franzmann and Alberti 2019).

### MC1 as a SG component

In this work, we focus on the characterization of MC1, an *Arabidopsis* Type I metacaspase. MC1 was previously shown to participate in immunogenic cell death and aging, although its mode of action remained obscure (Coll et al. 2010, 2014). Aging caused remobilization of MC1 from the soluble fraction to insoluble protein aggregates. Further, absence of MC1 caused overaccumulation of insoluble protein aggregates in aging cells, potentially leading to the observed accelerated senescence phenotype in *mc1* mutant plants. Aging, with its overall loss of proteostatic capacity, unveils mutant phenotypes linked to PQC failure that remain hidden in young cells due to their very efficient control of protein misfolding/aggregation. Thus, to investigate the role of MC1 in acute proteotoxic stress in cells with full proteostatic capacity, we used a simple, well-characterized system, heat stress on young seedlings, which lack persistent or aberrant condensates that accumulate as a result of aging (Gutierrez-Beltran et al. 2015; Kosmacz et al. 2018, 2019). We showed that upon heat stress, MC1 relocalizes into distinct cytoplasmic puncta that disappear during the recovery phase after stress removal (Figs. 1, A and B, and S2). These puncta correspond to SGs based on their dynamics (Figs. 1, A and B, and 4C), sensibility to CHX (Fig. 1, C and D), and colocalization/coimmunoprecipitation with SG markers in planta (Figs. 1, E and F, and S3) (Kosmacz et al. 2019; Gutierrez-Beltran et al. 2021). How much of the total MC1 protein pool localizes to SGs remains to be established.

Phase separation often drives the formation of SGs (Alberti and Carra 2018; Maruri-López et al. 2021; Allen and Strader 2022). The main features of proteins that form membraneless compartments such as SGs through LLPS are (i) presence of IDRs, (ii) complex domain organization, and (iii) their marginal solubility in the cell (Alberti and Carra 2018). MC1 is predicted to encompass 2 main IDR regions in its amino acid sequence, 1 coinciding with the N-terminal prodomain and the second near the C-terminus, known as the 360 loop (van Midden et al. 2021) (Fig. 2A). Further, these 2 regions are predicted to be highly insoluble and aggregation prone, in particular the 360 loop (Fig. 2B). In agreement with this prediction, MC1 became soluble only when both the prodomain and the 360 loop were simultaneously removed (Fig. 3). Interestingly, the highly hydrophobic 360 loop is only present in plant Type I metacaspases (van Midden et al. 2021). Type I metacaspases from protozoa and fungi do not possess this domain and, accordingly, they are soluble in vitro and they can be readily purified without removal of any domain (Lee et al. 2010; McLuskey et al. 2012).

In this regard, our data show that the 360 loop mediates MC1 recruitment into SGs during heat stress, as demonstrated by the drastic reduction in MC1-containing SGs formed in the 360 loopless mutant (Fig. 4). In contrast, in mutants lacking the prodomain, SGs still formed upon heat stress. However, these granules are less dynamic, showing reduced recovery after photobleaching (Fig. 4). This may indicate that the prodomain is involved in SG clearance. Considering that the prodomain has been proposed to act as a negative regulator of Type I metacaspase activity based on structural, as well as genetic data (Lee et al. 2010; McLuskey et al. 2012), it is tempting to speculate that the prodomain contains certain amino acids or motifs that may mediate recognition and degradation of MC1 by granulostasis, which has been shown to involve chaperone-mediated PQC, autophagy, or the ubiquitin–proteasome system (Alberti and Carra 2018). Interestingly, maize (*Zea mays*) MC1 has been shown to form puncta and colocalize with autophagosomes after heat stress when transiently expressed in *Nicotiana benthamiana* (Luan et al. 2021), indicating that autophagy may be a possible degradation route for MC1-containing SGs as shown for other aggregation-prone proteins (Munch et al. 2014; Jung et al. 2020).

### MC1 can specifically clear protein aggregates

From previous work in yeast (Lee et al. 2010; Hill et al. 2014) and plants (Coll et al. 2014), it was unclear how MC1 contributes to aggregate clearance. Work with the yeast metacaspase yMCA1 showed that upon heat stress and aging, the protein relocalizes to PQC condensates known as juxtanuclear quality control compartment (JUNQ) and IPOD (Hill et al. 2014). Here, we demonstrate that in plants, MC1 is recruited to SGs upon heat stress, but what is its function and how is it connected to aggregate clearance? First, we showed that mutants lacking MC1 accumulated higher levels of aggregate-prone aggregated proteins, such as polyQ-containing, HSP90, or actin, than WT plants under basal condition, a phenotype exacerbated after applying heat stress (Fig. 5A). Second, MC1 exhibited a strong and evolutionarily conserved capacity to degrade protein aggregates, as shown in various well-established systems in vitro (human protein) and in vivo (yeast and human cells) (Sant’Anna et al. 2016; Koyuncu et al. 2018; Llamas et al. 2022) (Figs. 5, S8, and S9). In particular, rMC1 showed an extraordinary capacity to clear aggregated TTR, a pathological form of the protein that causes a diversity of life-threatening pathologies (Saelices et al. 2015). MC1 proteolysis of TTR was prodomain and 360 loop independent but dependent on its catalytic activity. In fact, mutation of the catalytic cysteine in MC1 resulted in increased insolubility and self-aggregation of the protein, indicating a marked change in its biophysical properties. Still, it remains to be defined whether MC1-containing condensates are the physiological sites of protein aggregate clearance and what is the exact MC1 mode of action therein.

MC1 proteolytic activity was specifically directed toward aggregated forms of the protein, since monomeric TTR was not processed by MC1 (Fig. 5D). The protein aggregate-targeted behavior of MC1 could potentially be due to its slow kinetics. MC1 is an active protease, as shown by its ability to self-cleave and cleave its inhibitor serpin in vivo (Lema Asqui et al. 2018) and in vitro (Supplemental Fig. S4A). Here, we show that in addition to that, rMC1 can cleave metacaspase-specific synthetic substrates in vitro in the presence of calcium and neutral pH (Supplemental Fig. S5, B and C). However, compared to *Arabidopsis* Type II metacaspases, MC1 displays slower and/or less efficient protease activity toward typical metacaspase substrates (Vercammen et al. 2004; Hander et al. 2019; Zhu et al. 2020). We hypothesize that precisely this slower kinetics may favor its disaggregase activity, rather than a quicker protein-processing activity that could cleave monomeric forms of TTR. Notwithstanding, this newly discovered function of MC1 may inspire further research on protein disaggregases as an avenue for therapeutic intervention in age-related protein-misfolding diseases.

### Antiaging role of MC1: physiological implications of the role of MC1 in protein aggregate clearance

In previous work, we showed that plants lacking MC1 displayed accelerated senescence, which has been confirmed here using a different senescence-inducing system (Fig. 6A; Coll et al. 2014). Beyond that, here we present data demonstrating that overexpression of MC1 delays leaf senescence (Fig. 6A). The onset of senescence triggers the formation of cytoplasmic MC1-containing puncta that could also correspond to SGs, as they also contain TSN2 (Supplemental Fig. S11). Considering that in our system, we use dark as a trigger for senescence, 1 may speculate that the observed condensates are processing bodies (PBs) rather than SGs, since PBs have been shown to act as mRNA reservoirs under dark conditions (Jang et al. 2019). In contrast to SGs, PBs are constitutively present in the cell, but they have been shown to increase in number and size during stress conditions and exchange proteins and RNAs with SGs (Chicois et al. 2018; Gutierrez-Beltran et al. 2021; Solis-Miranda et al. 2023). A recent metaproteomic analysis of stress-induced condensates showing distinct protein signatures for SGs and PBs has revealed that MC1 is only present in SGs across various stress conditions (Solis-Miranda et al. 2023). Still, it will be interesting to determine the composition of MC1-containing condensates under natural and dark-induced senescence to define the degree of resemblance to stress-induced condensates.

Mutation of the conserved MC1 catalytic cysteine or removal of the 360 loop abolishes the observed senescence delay caused by overexpression of the protein. This indicates that MC1 proteolytic activity as well as its recruitment into SGs may be involved in this antiaging function of the protein. This prolife function of MC1 is evolutionarily conserved, as overexpression of yeast *MC1* can also extend replicative lifespan, a function partly dependent on the presence of an intact catalytic cysteine and attributed to a role in protein aggregate management as part of PQC (Hill et al. 2014).

An interesting question is why altering the levels of MC1 in knock-out mutants did not result in an obvious phenotypic effect compared to WT in response to heat (Supplemental Fig. S8), such as the 1 observed during senescence. A plausible explanation coming from the animal field is that young individuals/tissues/cells have multiple and very active misfolded protein clearance mechanisms, which can efficiently manage proteotoxicity ensued from stress situations, such as heat stress, even in the presence of mutations affecting PQC. In contrast, old individuals/tissues/cells experience a global decrease in proteostasis, uncovering the effect of mutations affecting PQC, which is the causative ground of many age-associated protein-misfolding diseases that appear late in life (Alberti and Hyman 2021).

**Figure 7.**
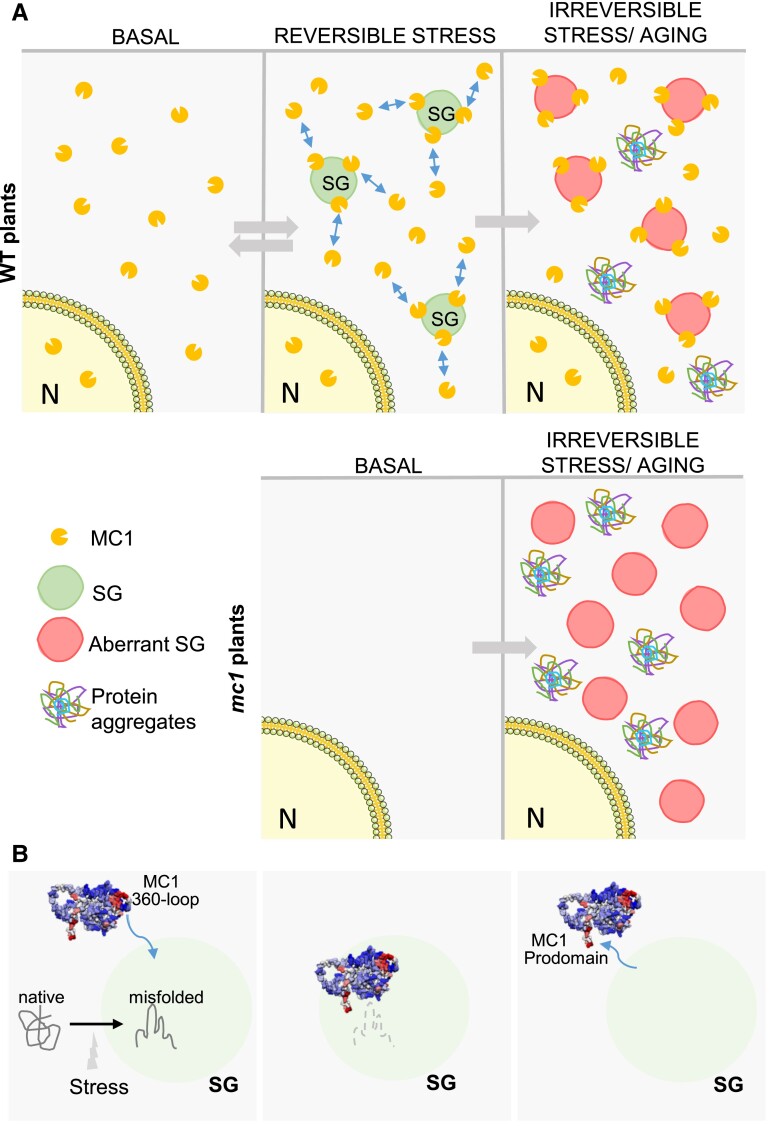
Working model on the role of MC1 in SGs. **A)** Upper panel (WT plants): Under basal conditions, no SGs are detectable and MC1 presents a diffuse nucleo-cytoplasmic localization pattern. Upon perception of an acute, reversible stress, MC1 is recruited to SGs where it hypothetically clears misfolded/aggregated proteins. Under chronic or irreversible stress, the proteostatic capacity of the cell is surpassed and toxic protein aggregates that cannot be cleared start accumulating in the cytoplasm. Lower panel (*mc1* mutant plants): Plants devoid of MC1 cannot cope as WT with proteotoxic stress. Any stress may result in accumulation of protein aggregates that over time manifest as the observed accelerated senescence phenotype. **B)** MC1 is recruited to SGs via its 360 loop. Once in SGs, it clears aggregated proteins via its disaggregase activity. Release of MC1 from SGs is dependent on the prodomain. Nuclear schemes in **A)** and **B)** were obtained from Bioicons/Servier/license CC BY 3.0. **C)** MC1 is recruited to SGs via its 360 loop. Once in SGs, it clears aggregated proteins via its disaggregase activity. Release of MC1 from SGs is dependent on the prodomain.

All considered, a plausible hypothesis is the following (Fig. 7): (i) proteotoxic stress, such as heat stress, triggers the formation of SGs; (ii) MC1 is recruited to SGs via the 360 loop; and (iii) once there, MC1 participates in protein clearance via its proteolytic activity to help dissolving the granules. In favor of this hypothesis, removal of the catalytic cysteine of MC1 does not affect recruitment into SGs, but it alters aggregate clearance.

## Materials and methods

### Materials used and growth conditions

All experiments were performed using *A. thaliana* Col-0 ecotype. Lines used for this work are listed in Supplemental Data Set 1. The single mutant *mc1* has been previously described (GK-096A10; Coll et al. 2010). All seeds were surface sterilized with 35% NaClO for 5 min and washed 5 times for 5 min with sterile dH_2_O. Sterile seeds were sown in solid ½ MS medium with vitamins and stratified 48 h at 4 °C. Plants were grown vertically under long day (LD) conditions (16-h light/8-h dark) at 22 °C. For dark-induced senescence studies, 1-wk-old seedlings were transferred into soil and grown for an additional 2 wk under LD conditions.

The following transgenic lines in *mc1* background were used for SG visualization and dark-induced senescence studies: *Pro35S:MC1-GFP*, *Pro35S:MC1C220A-GFP*, *Pro35S:ΔNMC1-GFP*, *Pro35S:MC1Δ360loop-GFP*, *ProMC1:MC1-GFP*, and *ProMC1:MC1C220A-GFP* (Supplemental Data Set 1). Transgenic *Col-0* lines expressing *Pro35S:GFP-TCTP* (Gutierrez-Beltran et al. 2021) and *Pro35S:MC1-RFP* or *ProTSN2:TSN2-GFP* (Gutierrez-Beltran et al. 2021) and *Pro35S:MC1-RFP* were used for SG colocalization experiments. Additionally, *Col-0* WT and *mc1* mutant plants expressing *35S:RFP-Rbp47* were used for FRAP analysis.

### Plasmid construction

All constructs and primers used in this study are described in Supplemental Data Sets S2 and S3, respectively. To generate *Pro35S:MC1-GFP*, *Pro35S:MC1-RFP*, *Pro35S:ΔNMC1-GFP*, *Pro35S:MC1Δ360loop-GFP*, *ProMC1:MC1-GFP*, and *ProMC1:MC1C220A-GFP* constructs, the coding sequence and native promoter (∼1 kb) of *Arabidopsis MC1* (*AT1G02170*) were amplified from Col-0 cDNA and genomic DNA, respectively. Plasmids were assembled through GreenGate cloning (Lampropoulos et al. 2013). To generate *Pro35S:RFP-Rbp47* and *Pro35S:RFP-TSN2*, the coding sequences of *Rbp47 (AT3G19130*) and *TSN2 (AT5G61780*) were amplified from *Col-0* cDNA and cloned into pGWB655 following the Gateway strategy (Karimi Inzé and Depicker 2002).

For purification of rMC1, the MC1 coding sequences lacking the 360 loop and with or without the prodomain (MC1Δ360 or ΔNMC1Δ360/rMC1) (Fig. 2) were synthesized (Twist Bioscience) with codon optimization for expression in *E. coli*. Synthetic sequences contained NdeI and XhoI restriction sites at the 5´ and 3´ ends of the sequence, respectively. Both synthetic genes and destination vector pET28 b(+) were cut with NdeI and XhoI and subsequently ligated so that an N-terminal 6xHis tag precedes the start site of the MC1 variants. QuickChange Site-Directed Mutagenesis (Agilent Technologies) was used to cause point mutations in the catalytic site of rMC1.

To complement the yeast metacaspase mutant strain *ymca1Δ* with *Arabidopsis* MC1 (AT1G02170), we constructed a gene replacement cassette by PCR-directed homologous recombination (Gardner and Jaspersen 2014). The cassette consisted of 3 fragments: (i) yeast 5′ flanked with N-acetyltransferase (NAT) resistance gene and a glyceraldehyde-3-phosphate dehydrogenase (GPD) constitutive promoter, (ii) *Arabidopsis MC1* gene fused to a C-terminal HA tag, and (iii) 3′ flanking of the yeast *yMCA1* gene. These 3 fragments were PCR amplified and fused into the final recombinant DNA by using the double-joint PCR method as previously described (Yu et al. 2004). The resulting DNA product (*NATNT2:pGPD:AtMC1-HA*) was transformed into *the ymca1Δ* mutant strain KanMX4.

### Protoplasts and plant transformation

*Arabidopsis* protoplasts were obtained as previously described (Truskina et al. 2020). In short, leaves from 3-wk-old plants were collected and digested in an enzyme solution (1% cellulose R10, 0.25% macerozyme R10, 0.4 M mannitol, 10 mM CaCl_2_, 20 mM KCl, 0.1% BSA, and 20 mM MES at pH 5.7) for 1 or 2 h. Protoplasts were collected through a 70-micron strainer, washed twice with ice-cold W5 solution (154 mM NaCl, 125 mM CaCl_2_, 5 mM KCl, 5 mM glucose, and 2 mM MES at pH 5.7), and incubated on ice for 30 min. The protoplasts were then resuspended in mannitol-magnesium solution (0.4 M mannitol, 15 mM MgCl2, and 4 mM MES at pH 5.7) at a final concentration of 2.5 × 10^5^ cells/ml. To transform the protoplasts, 30 *μ*g of the appropriate plasmid were mixed with 200 *μ*l of protoplast solution. Immediately, 210 *μ*l of PEG solution (40% PEG 4000, 0.2 M mannitol, and 0.1 M CaCl_2_) was added and the protoplasts were incubated for 5 min at room temperature and then washed twice in W5 solution. The protoplasts were resuspended in 500 *μ*l of the W5 solution and incubated for 24 h in 16-h light/8-h dark growth chamber. Before imaging, the protoplasts were incubated at 39 °C for 40 min in a hot air incubator.

*Arabidopsis* plants were transformed as described previously through the *Agrobacterium tumefaciens*–mediated floral-dip method (Clough and Bent 1998).

### Yeast strains and spot dilution assays

Yeast media preparation and molecular biology techniques were carried out using standard methods (Lázaro-Silva et al. 2015). All experiments were done using the genetic background of *Saccharomyces cerevisiae* strain BY4741 (Supplemental Data Set 4). To test the capacity of each yeast strain to remove misfolded proteins, yeast cells were transformed with the plasmid pFE15 encoding the fusion construct ΔssCL∗myc (Supplemental Data Set 2; Eisele and Wolf 2008). Growth phenotypes were assessed with spot dilution assays. Ten-fold serial dilutions were made, ranging from undiluted to a 10^4^ dilution. Five microliters of each dilution were spotted onto the corresponding selective media (-ura or -ura -leu plates), and plates were incubated for at least 3 d before images were taken.

### HEK293T cells transfection and protein extraction

The *MC1* (*AT1G02170*) gene was codon optimized for expression in animal cells and synthetized (Twist Bioscience). To generate *ProCMV:GFP-MC1*, the synthetic gene was cloned in the pDEST-CMV-N-GFP vector by Gateway technology (Addgene). *ProCMV:mRFP-Q74* (Balaji et al. 2022), *ProCMV:GFP-MC1*, and *ProCMV:GFP* (Llamas et al. 2022) were used for transfection of HEK cells (CRL-1573) (Supplemental Data Set 5) following the protocol described in (Llamas et al. 2022). After 72 h of incubation, cells were lysed in nondenaturing native lysis (300 mM NaCl, 100 mM HEPES pH 7.4, 2 mM EDTA, and 2% Triton X-100) supplemented with 1× plant protease inhibitor (Merck), scraped from the tissue culture plates, and homogenized through a syringe needle (27G). Samples were centrifuged at 10,000 × *g* for 10 min at 4 °C and supernatant was collected. Protein concentration was determined with the Pierce BCA Protein Assay Kit (Thermo Fisher).

### Microscopy analysis

Microscopy images were acquired with an Olympus FV1000 inverted confocal microscope with a 60×/water objective. For detection of fluorescent signals, GFP was excited at 488 nm and mRFP at 543 nm.

Root meristem cells from 5-d-old seedlings vertically grown under LD conditions (16-h light/8-h dark) at 22 °C were used to determine protein subcellular localization. For heat stress treatment, 5-d-old seedlings were transferred to a hot air incubator at 39 °C and incubated for 40 min. For CHX treatment, 5-d-old seedlings were incubated in ½ MS liquid medium with 200 ng/*µ*l CHX for 30 min. A number of granules were quantified with ImageJ using the SiCE spot detector macro for automatic quantification (Bayle et al. 2017).

### FRAP

The assay was performed as described previously (Moschou et al. 2013). Five-day-old seedlings grown vertically in LD conditions were incubated for 40 min at 39 °C in a hot air incubator. During analyses, the Olympus FV1000 software was set up for the acquisition of 2 prebleach images, 1 bleach scan, and 30 postbleach scans. A region 2 *µ*m in diameter was bleached using a laser intensity of 100% at 488 nm. Prebleach and postbleach scans were at the minimum possible laser power. A zoom factor of 5 was used.

Analyses of fluorescence intensities during FRAP were performed in the bleached regions. One region of interest outside of the bleached area was also measured to serve as the background. The background values were subtracted from the fluorescence recovery values, and the resulting values were normalized by the first postbleach time point. Initial signal recovery (%) = 100 × (Ifinal, postbleach − Iinitial, postbleach)/(Iprebleach − Iinitial, postbleach), where I is the normalized signal intensity (relative to the background intensity).

### Protein purification

*E. coli* OverExpress C41 (DE3) Chemically Competent Cells from BioCat GmbH (Heidelberg, Germany) or *E. coli* BL21 strain containing the pBB542 vector (de Marco et al. 2007) were transformed with expression plasmids and grown in either autoinduction media or LB, respectively. Cells were grown first at 37 °C with continuous shaking until OD_600_ reached 0.6 and then transferred to 25 °C for overnight growth. In the case of expression in *E. coli* Chaperone Competent Cells BL21, isopropyl β-D-1-thiogalactopyranoside (IPTG) at 1 mM concentration was added to 400-ml cell cultures when transferred to 25 °C to induce protein expression. The pellet from overnight cultures was resuspended in 20 mM HEPES, pH 7.5, and 500 mM NaCl and sonicated on ice. A centrifugation of lysates at 25,000 × g for 20 min was performed to remove cell debris and insoluble proteins. Soluble lysate was filtered through 0.45 *µ*M sterile filters and loaded into a 5-ml nickel ion HisTrap purification column (Cytiva, Marlborough, MA, USA). Washes of the columns were performed with 20 mM HEPES, pH 7.5, 500 mM NaCl, and 20 mM imidazole. Elution of proteins was performed by increasing imidazole concentrations up to 250 mM. The cleanest elutions were concentrated using Amicon filters and loaded onto a Superdex 75 size-exclusion chromatography column (GE Healthcare Life Sciences, Chicago, IL, USA) connected to an AKTA FPLC system. The Superdex 75 column was equilibrated in 20 mM HEPES, pH 7.5, and 500 mM NaCl. A flow rate of 0.75 ml per minute was used to separate proteins. Samples belonging to the most prominent peaks were kept and loaded onto an SDS–PAGE gel to verify the purity of the samples. MC4 was provided by F. van Breusegem and it is described in Vercammen et al. (2004).

### Enzymatic activity assays

Protease activity was measured by quantification of the fluorescence intensity released from the AMC group of the fluorogenic substrate Z-FR-AMC (PeptaNova, Sandhausen, Germany) at 383- and 455-nm excitation and emission wavelengths, respectively, in a Tecan Infinite M200 Microplate Reader System (Männedorf, Switzerland). All proteolytic assays were performed in 20 mM HEPES (pH 7.0) containing 150 mM NaCl, varying CaCl_2_ concentrations and 5 mM DTT. For estimation of pH optima, buffers containing 100 mM acetate (pH 4 to 5.5), 100 mM MES (pH 6 to 6.5), 100 mM HEPES (pH 7.0 to 8.0), 100 mM Tris (pH 8.5 to 9), and 100 mM CAPS (pH 9.5 to 11) were used. 0.2 *μ*g of recombinant protease was used and the concentration of fluorogenic substrates was 5 *μ*M.

### Preparation of TTR aggregates

TTR was expressed and purified following previously described procedures (Pinheiro et al. 2021). Briefly, TTR aggregation was induced by mixing 7 *μ*M purified TTR with an equal volume of 400 mM sodium acetate, 200 mM KCl, and pH 4.4, obtaining a final TTR concentration of 3.5 *μ*M. Samples were incubated for 72 h at 37 °C in quiescent conditions. Aggregated samples were centrifuged at 20,000 × *g* for 1 h to recover the insoluble material that was subsequently resuspended in 20 mM HEPES, 150 mM NaCl, and pH 7.5 to a concentration of 100 *μ*M.

### In vitro disaggregation assay

End-point disaggregation reactions were performed by coincubating TTR aggregates at a concentration of 7 *μ*M with 0.25 mg/ml of protease at 37 °C in presence of 5 mM DTT and 5 mM CaCl_2_. Protease disaggregation was monitored using sample turbidity, SDS–PAGE, and TEM.

### Turbidity assay

Sample turbidity was monitored as an indicator of the amount of aggregated material using synchronous light scattering. The spectra were recorded in a JASCO Spectrofluorometer FP-8200 with an excitation wavelength of 360 nm and emission range from 340 to 380 nm. Excitation and emission bandwidth were set to 5 nm. The light scattered at 360 nm was used as a measure of turbidity.

### Protein extraction and immunoblotting

Five hundred milligrams of leaf material were mixed with 2 ml of extraction buffer (50 mM HEPES pH 7.3, 150 mM NaCl, 0.5% Nonidet P-40, 10% glycerol, 1 mM EDTA pH 8, 5 mM DTT, 1% PVPP, and 1× Protease Inhibitor Cocktail [Sigma, P599]) and centrifuged for 10 min at 14,000 × *g* at 4 °C; 5× Laemmli sample buffer was added to 100-*µ*l supernatant and boiled for 5 min. Equal amounts of supernatant were loaded on 12% SDS–PAGE gels. Antibodies used for immunoblotting were as follows: α-GFP-HRP (1:5,000 Miltenyi Biotec), α-RFP-HRP (1:5,000 Abcam), α-myc (1:10,000, Sigma-Aldrich), α-actin (dilution 1:5,000, Agrisera), α-Hsp90-1 (1:2,000 Abcam), and α-polyQ (1:1,000 Merck).

### Filter trap assay

Protein extracts were obtained with native lysis buffer (300 mM NaCl, 100 mM HEPES pH 7.4, 2 mM EDTA, and 2% Triton X-100) supplemented with EDTA-free protease inhibitor cocktail. When processing plant protein extracts, 1× plant protease inhibitor (Merck) was added to native lysis buffer. In experiments with HEK cells, cells were homogenized by passing 7 times through syringe needle (27 G). Cellular debris was removed by several centrifugation steps at 8,000 × *g* for 10 min at 4 °C. Supernatant was recollected and protein concentration determined with the Pierce BCA Protein Assay Kit (Thermo Fisher). A cellulose acetate membrane filter (GE Healthcare Life Sciences) was placed in a slot blot apparatus (Bio-Rad) coupled to a vacuum system. The membrane was equilibrated with 3 washes with equilibration buffer (native buffer supplemented with 0.5% SDS). Approximately 150 *µ*g of protein extract was supplemented with SDS at a final concentration of 0.5% and loaded and filtered through the membrane. Then, the membrane was washed 3 times with 0.2% SDS. The membrane was blocked in 3% BSA in TBST for 30 min followed by 3 washes with TBST. The membrane was incubated with indicated antibody and then washed 3 times for 5 min and incubated with secondary antibodies in TBST 3% BSA for 30 min. The membrane was developed using an Odyssey DLx (Licor). Extracts were also analyzed by SDS–PAGE and western blotting to determine loading controls.

### Heat treatments

Thermotolerance assays were performed using 5-d-old seedlings. Seedlings were grown at 22 °C for 5 d, put in a hot-air incubator set at 37 °C for 90 min, put in a growth chamber set at 37 °C for 90 min, incubated at 45 °C for 90 min, and allowed to recover at 22 °C for 8 d. The percentages of seedlings in different phenotypic classes were calculated based on results from 3 biological replicates. In each biological replicate, at least 50 seedlings were used for each genotype.

### Dark-induced senescence assay

Leaves 5 and 6 of 3-wk-old plants grown in LD conditions were covered with aluminum foil for 8 d (Li et al. 2016). Control plants kept without covered leaves were grown in parallel.

### Chlorophyll analysis

Covered and uncovered leaves from 3 different plants were snap frozen in liquid nitrogen and ground with TissueLyser II (QIAGEN). A 50-mg aliquot of crushed leaf material was mixed with 1.5 ml of 80% prechilled acetone and thoroughly mixed for 5 min. Samples were centrifuged at 20,000 × *g* for 1 min, and the supernatant was transferred to spectrophotometer cuvettes. Chlorophyll was then quantified at 663 and 646 nm with a spectrophotometer (UV-2600, Shimadzu) as previously described (Lichtenthaler and Wellburn 1983).

### Pulse amplitude modulation (PAM) fluorometric measurements

After 30 min of dark adaptation, the kinetics of chlorophyll fluorescence in whole rosettes were monitored by measuring F0 in the dark and Fm with initial saturation pulse using Imaging PAM M-series, MAXI version device (Walz). Fv/Fm and Fv′/Fm′ (PSII efficiency) ratio for the maximum quantum efficiency upon dark and light conditions was calculated according to the manufacturer's instructions.

### RT-qPCR analysis

The Maxwell RSC Plant RNA kit (Promega) was used to isolate RNA from covered and uncovered leaves from 3 different plants. Two micrograms of RNA were reverse transcribed into cDNA with the High-Capacity cDNA Reverse Transcription Kit with RNase inhibitor (Applied Biosystems). RT-qPCRs were performed with LightCycler SYBRgreen I master (Roche) in a LightCycler 480 System (Roche). *SGR1* (*AT4G22920*) expression was normalized to expression of ACT8 (AT1G49240), and data were analyzed using the ΔΔCT method. Primers for RT-qPCR used in this study were previously described and are listed in Supplemental Data Set 3 (Ueda et al. 2020).

### Bioinformatic analyses

IDRs of MC1 were predicted using the D2P2 database (D^2^P^2^, http://d2p2.pro/; Oates et al. 2013) and DISOPRED3 (http://bioinf.cs.ucl.uk/psipred; Jones and Cozzetto 2015). LLPS predisposition was evaluated using the PSPredictor tool (http://www.pkumdl.cn:8000/PSPredictor/; Chu et al. 2022). Analysis of the aggregation propensity of MC1 amino acids was performed with A3D (http://biocomp.chem.uw.edu.pl/A3D/; Zambrano et al. 2015) using as input file the AlphaFold2 (https://alphafold.ebi.ac.uk/; Jumper et al. 2021) predicted MC1 structure.

### Statistical analysis

All quantification analyses and statistical tests were performed with R software. *T*-test was used to compare the significance of differences between 2 experimental groups. For comparing the significance of differences between multiple experimental groups, 1-way ANOVA or Kruskal–Wallis 1-way analysis were performed as indicated in each experiment. Different letters show statistically significant differences between samples. Statistical analysis results are listed in Supplemental Data Set 6.

### Accession numbers

Sequence data for the genes described in this study can be found in the TAIR database (https://www.arabidopsis.org) and NCBI under the following accession numbers: *MC1* (*AT1G02170*), *RBP47* (*AT3G19130*), *TCTP* (*AT3G16640*), and *yMCA1* (*Q08601*).

## Supplementary Material

koad172_Supplementary_Data

## Data Availability

The data that support the findings of this study are available from the corresponding author upon reasonable request.

## Supplemental data

The following materials are available in the online version of this article.

**Supplemental Figure S1.** The size of MC1-containing SGs changes over time.

**Supplemental Figure S2.** MC1-GFP expressed under the control of its own promoter relocalizes to cytoplasmic condensates upon heat stress.

**Supplemental Figure S3.** MC1 colocalizes with SG markers in protoplasts upon heat stress.

**Supplemental Figure S4.** Production and purification of rMC1 in *E. coli* cells.

**Supplemental Figure S5.** rMC1 is a proteolytically active enzyme and behaves as a canonical Type I metacaspase.

**Supplemental Figure S6.** Immunoblot analysis of transgenic plants expressing the different MC1 variants.

**Supplemental Figure S7.** MC1 mediates the association of Rbp47b with SGs.

**Supplemental Figure S8.** *mc1* knock-out mutants do not display thermotolerance or total protein accumulation differences compared to WT.

**Supplemental Figure S9S.** MC1 participates in the clearance of terminally misfolded proteins.

**Supplemental Figure S10.** Relative expression of the senescence marker *SGR1* (*AT4G22920*).

**Supplemental Figure S11.** Dark-induced senescence results in the formation of MC1-containing cytoplasmic condensates.

**Supplemental Data Set 1.** *Arabidopsis* lines used in this study.

**Supplemental Data Set 2.** List of plasmids used in this study.

**Supplemental Data Set 3.** List of primers and synthetic sequences used in this study for genotyping and cloning.

**Supplemental Data Set 4.** Yeast strains used in this study.

**Supplemental Data Set 5.** Other materials used in this study.

**Supplemental Data Set 6.** Table for statistical analysis.
