# Supplementary material for: Arabidopsis metacaspase MC1 localizes in stress granules, clears protein aggregates, and delays senescence
Source: Plant Cell. 2023 Jul 1;35(9):3325–44. doi: 10.1093/plcell/koad172 (PMC10473220; doi:10.1093/plcell/koad172)
Supplement: koad172_Supplementary_Data [file koad172_supplementary_data.zip › Supplementary figures_3rdSbmission.pdf]

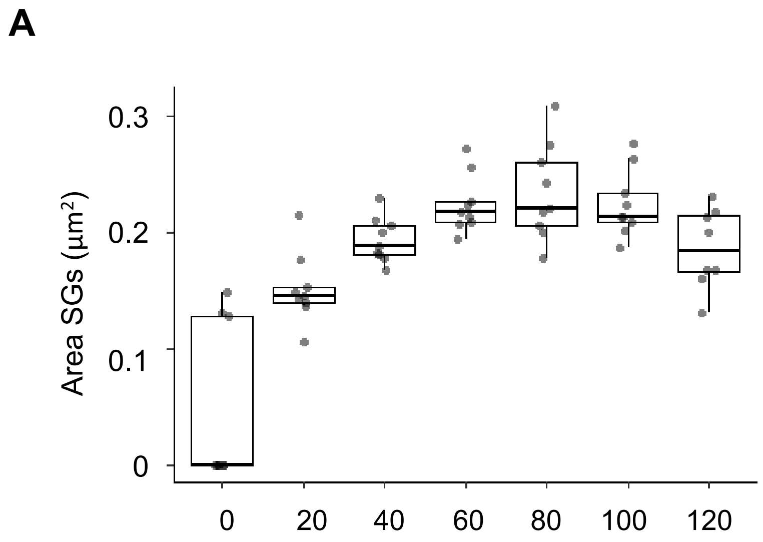

Figure S1. The size of MC1-containing SGs changes overtime (Supports Figure 1).
A) Five-day-old Arabidopsis *mc1* mutant seedlings expressing *35SMC1:MC1-GFP* were heat-stressed at 39ºC for 40 min. Upper and lower box boundaries represent the first and third quantiles, respectively; horizontal lines mark the median and whiskers mark the highest and lowest values. Three independent experiments, each containing measurements from approximately 90 cells from the root meristem, were performed.

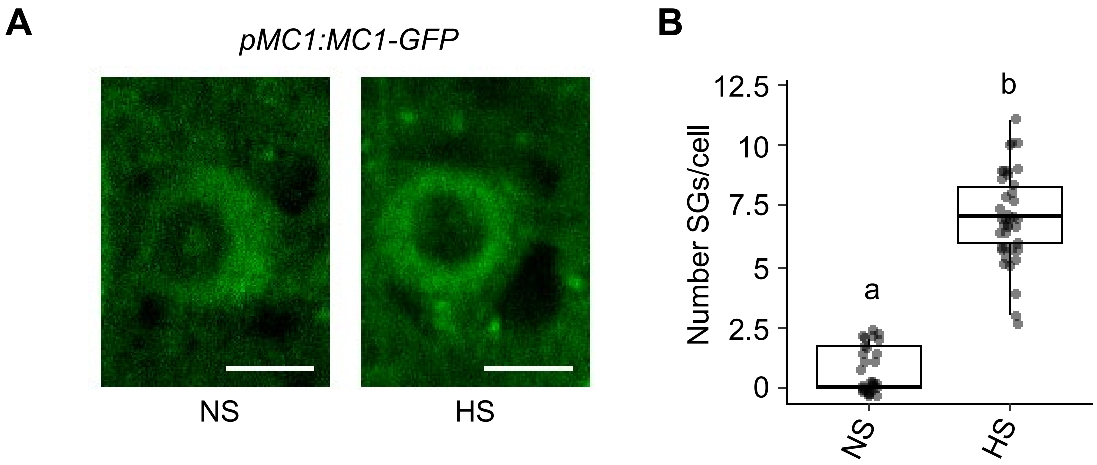

Figure S2. MC1-GFP expressed under the control of its own promoter re-localizes to cytoplasmic condensates upon heat stress (Supports Figure 1).

A) Five-day-old Arabidopsis *mc1* mutant seedlings expressing *ProMC1:MC1-GFP* were heat-stressed at 39ºC for 40 min. Images of root tips were taken before (NS) and after being subjected to heat stress (HS) Bars = 5 µm.

B) Quantification of MC1-GFP foci in the experiment shown in A). Upper and lower box boundaries represent the first and third quantiles, respectively; horizontal lines mark the median and whiskers mark the highest and lowest values. Three independent experiments, each containing five individual measurements, were performed. Means with different letters are significantly different at $P < 0.05$ (T-test).

A

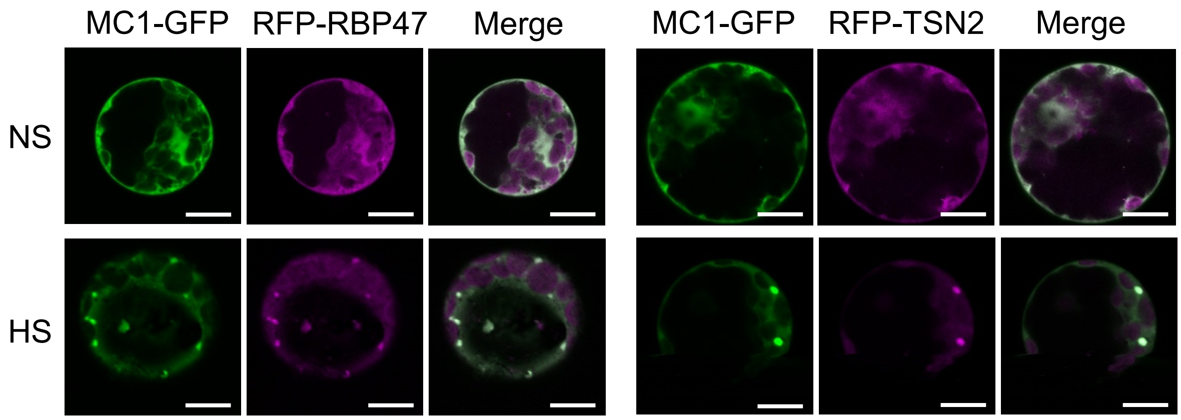

B

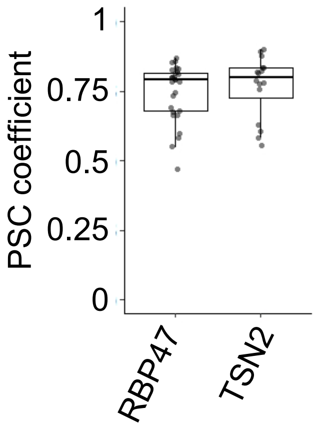

Figure S3. MC1 co-localizes with stress granule markers in protoplasts upon heat stress (Supports Figure 1).

A) Co-localization analysis using leaf protoplasts of 3-week-old *mc1 Pro35S:MC1-GFP* Arabidopsis plants transiently co-expressing *Pro35S:RFP-RBP47* or *Pro35S:RFP-TSN2.* Images were taken in control (NS) conditions or after heat-stressing (HS) the protoplasts.

B) Pearson coefficient of co-localization of RFP-RBP47 or RFP-TSN2 and MC1-RFP. Upper and lower box boundaries represent the first and third quantiles, respectively; horizontal lines mark the median and whiskers mark the highest and lowest values. Three independent experiments, each containing at least five individual measurements, were performed.

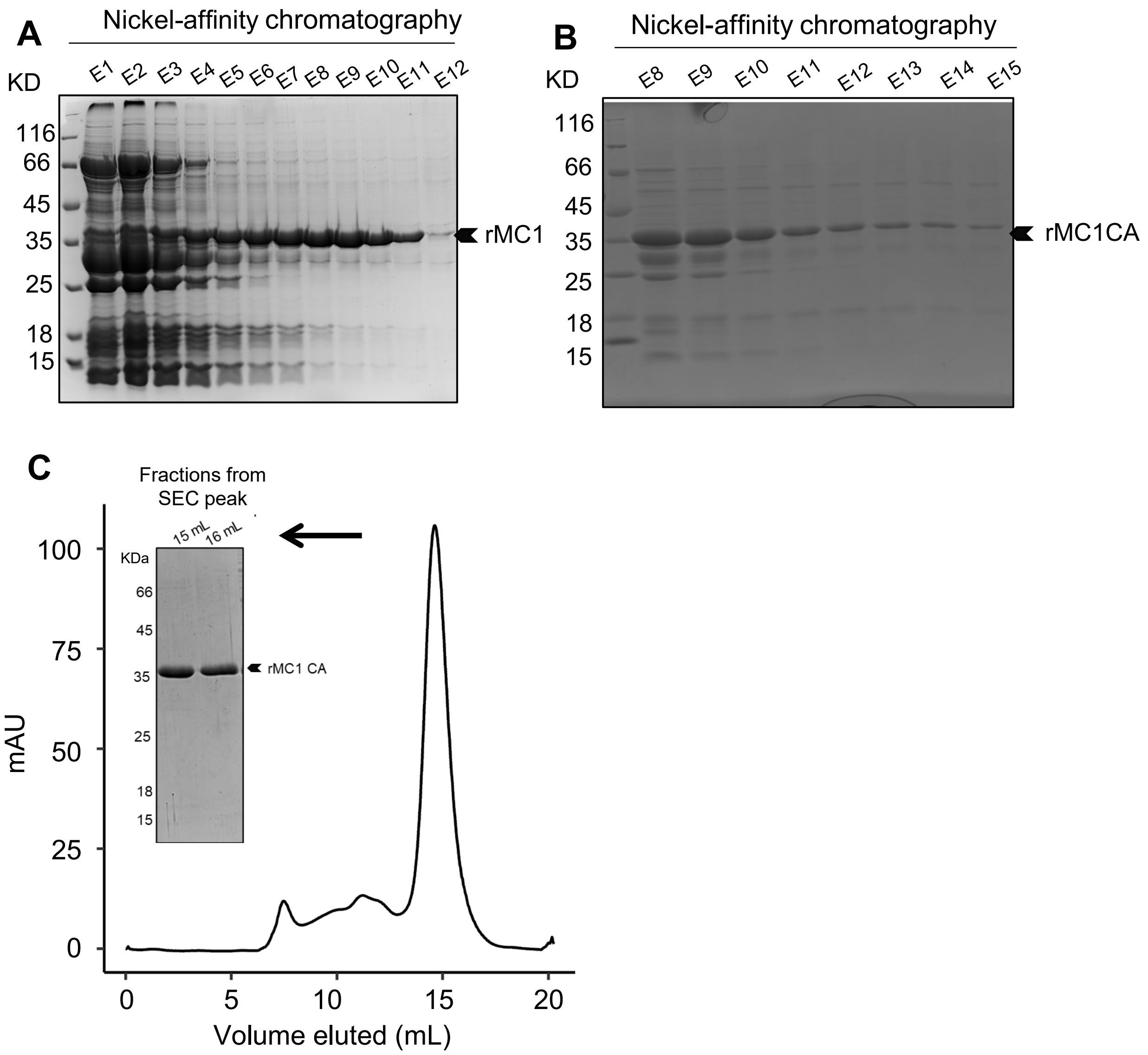

Figure S4. Production and purification of recombinant MC1 in *Escherichia coli* cells (Supports Figure 3).

A and B) SDS-PAGE Coomassie-stained gels of eluted fractions after nickel-affinity chromatography of *Escherichia coli* soluble lysates expressing either rMC1 (A) or rMC1CA (B). Arrow indicates expected molecular weight of rMC1.

A) Size-exclusion chromatography (SEC) from concentrated eluates shown in B. The inlet shows an SDS-PAGE Coomassie-stained gel of fractions 15 ml and 16 mL of the eluted volume from a Superdex 75 column.

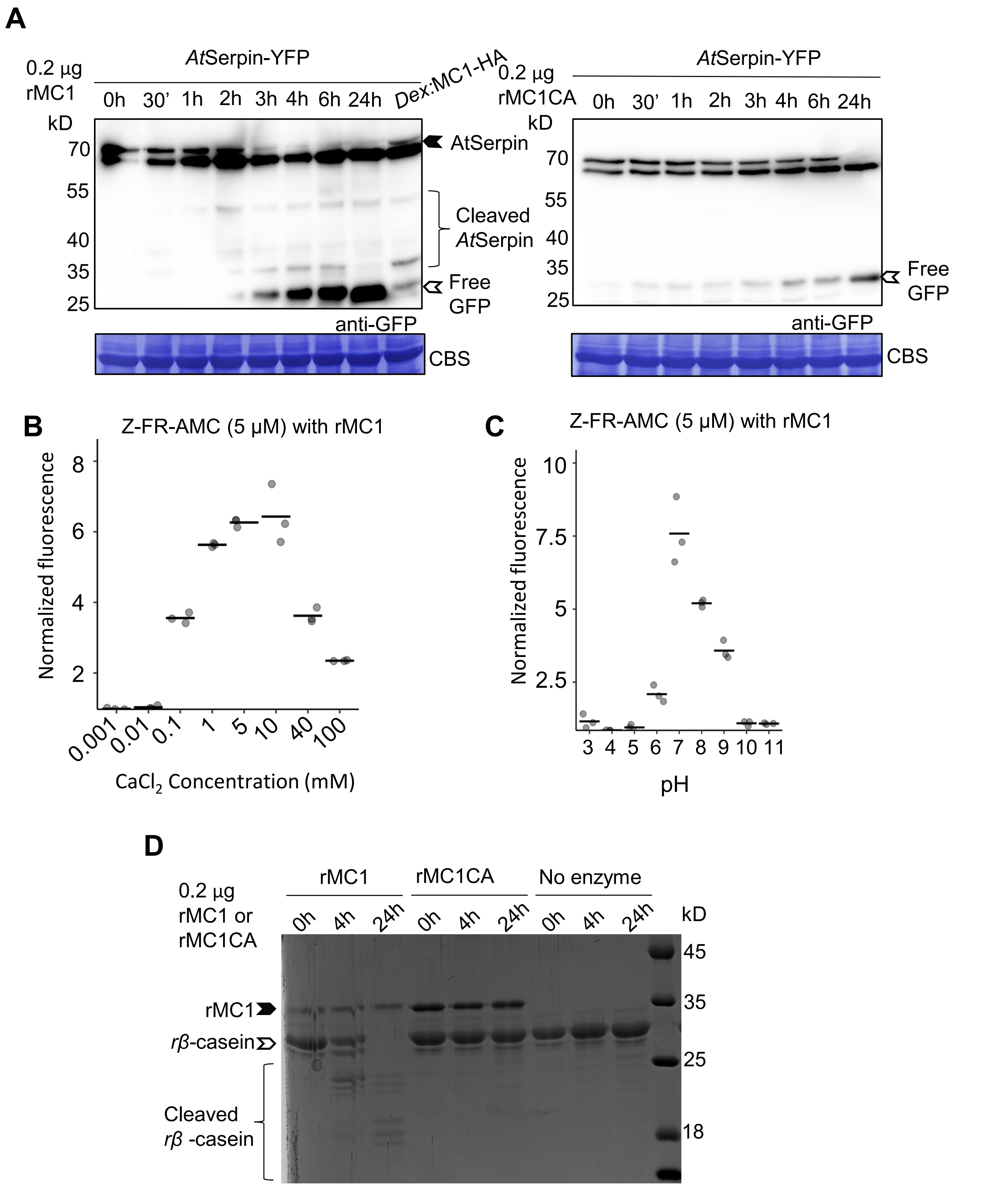

Figure S5. rMC1 is a proteolytically active enzyme and behaves as a canonical type I metacaspase (continues in the next page) (Supports Figure 3).

Figure S5. rMC1 is a proteolytically active enzyme and behaves as a canonical type I metacaspase (Supports Figure 3).

A) Immunoblot analysis of *Nicotiana benthamiana* protein extracts transiently expressing Serpin-YFP incubated with either 0.2 µg rMC1 or 0.2 µg rMC1CA for the indicated times (hours) at room temperature. Extracts from *N. benthamiana* plants co-expressing *At*Serpin-YFP and MC1 fused to HA (Dexamethasone:MC1-HA) were included in the experiment as a positive control for Serpin1 cleavage (Lema Asqui *et al.*, 2018). Coomassie blue staining of immunoblotted membranes (CBS) are shown as loading controls.

C-D) Activity of the fluorogenic substrate (Z-FR-AMC; 5 µM) when incubated with 0.2 µg of rMC1 under different concentrations of $CaCl_2$ **(C)** or different pH in 5 mM $CaCl_2$ (D).

E) SDS-PAGE Coomassie-stained gel of 2 µg *rβ*–casein incubated with 200 µg of either rMC1 or rMC1CA for 0, 4 or 24 h.

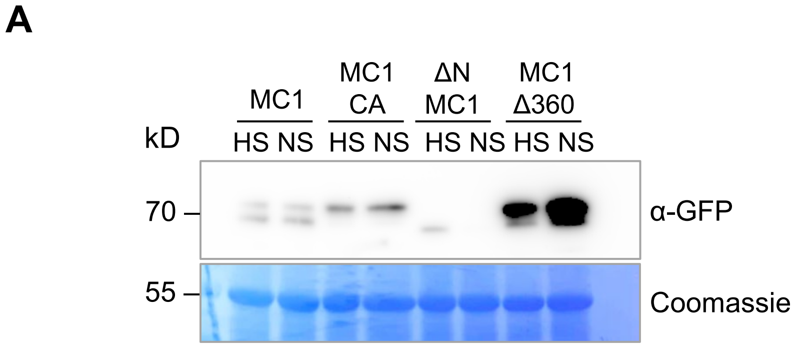

Figure S6. Immunoblot analysis of transgenic plants expressing the different MC1 variants (Supports Figure 4).

SDS-PAGE of protein extracts from five-day-old *Arabidopsis mc1* seedlings expressing *Pro35S:MC1-GFP, Pro35S:MC1C220A-GFP*, *Pro35S:ΔNMC1-GFP* or *Pro35S:MC1Δ360loop-GFP* grown in control conditions (NS) or subjected to heat stress (HS, 40 min at 39ºC). GFP antibody was used to detect MC1-GFP and Coomassie was used as loading control.

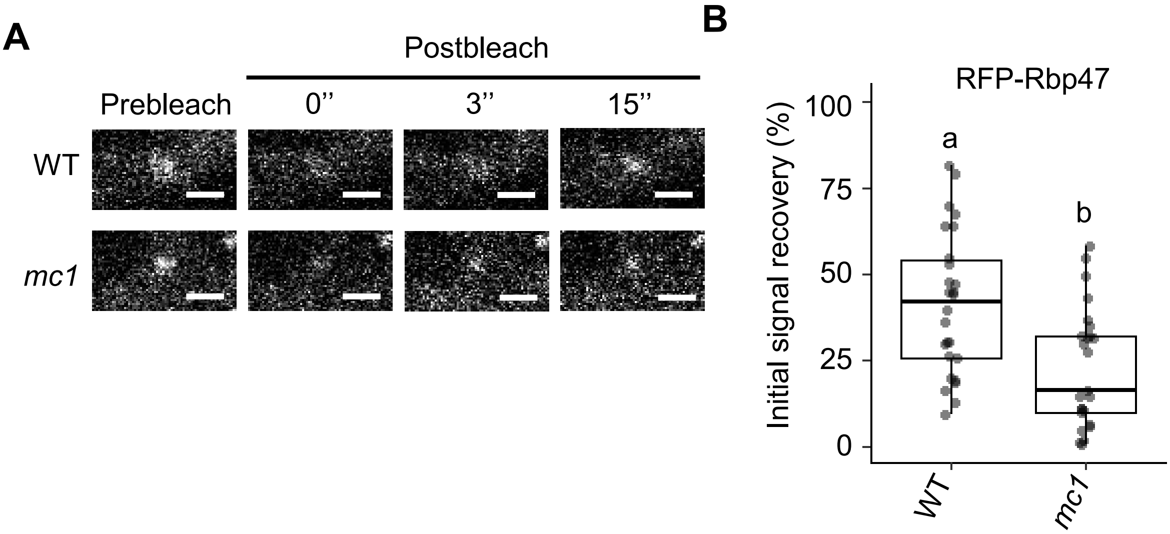

Figure S7. MC1 mediates the association of Rbp47b with SGs (Supports Figure 4).

A) Selected time frames (prebleach and 0, 3, and 15 seconds after bleaching) from FRAP analysis of RFP-Rbp47 foci formed upon heat stress (40 min at 39°C) in root tip cells of WT or *mc1* seedlings expressing *Pro35S:RFP*-Rbp47. Bars = 2µm.

B) Initial signal recovery (%) of the experiment shown in B. Upper and lower box boundaries represent the first and third quantiles, respectively; horizontal lines mark the median and whiskers mark the highest and lowest values. Three independent experiments, each containing at least eight individual measurements, were performed. Means with different letters are significantly different at $P < 0.05$ (one-way ANOVA).

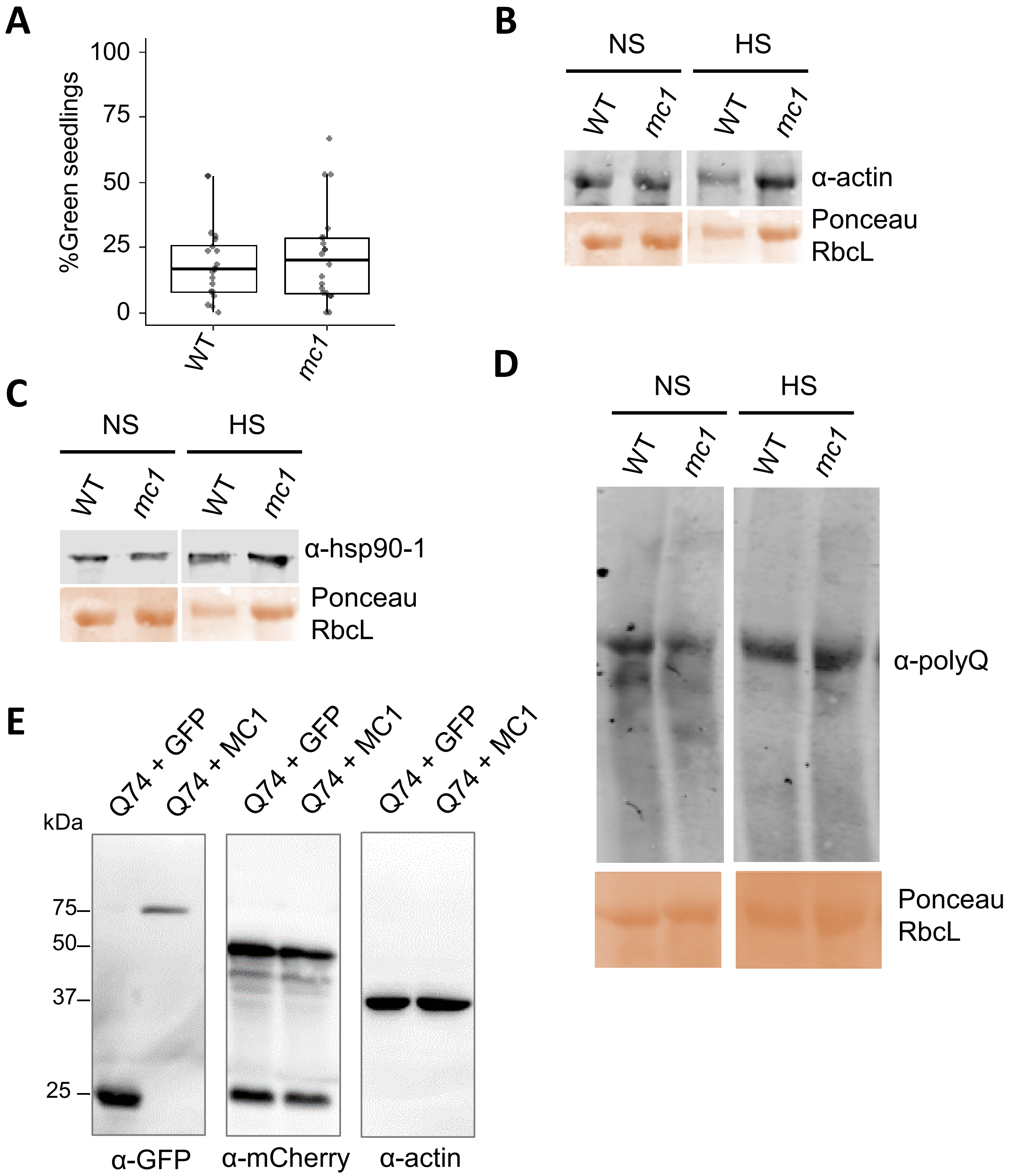

Figure S8. *mc1* knock-out mutants do not display thermotolerance or total protein accumulation differences compared to WT (continues next page) (Supports Figure 5).

Figure S8. *mc1* knock-out mutants do not display thermotolerance or total protein accumulation differences compared to WT (Supports Figure 5).

A) Five-day-old WT or *mc1* Arabidopsis seedlings were subjected to heat stress (HS, 90 min at 37ºC, 90 min at 22ºC and 90 min at 45ºC) followed by incubation at 22ºC for 7 days. Data represents the percentage of green seedlings. Upper and lower box boundaries represent the first and third quantiles, respectively; horizontal lines mark the median and whiskers mark the highest and lowest values. Ten independent experiments were performed. Means with different letters are significantly different at $P < 0.05$ (one-way ANOVA).

B, C and D) SDS-PAGE of protein extracts from five-day-old *Arabidopsis mc1* or WT seedlings in control conditions (NS) or subjected to a severe heat stress (HS, 90 min at 37ºC, 90 min at 22ºC and 90 min at 45ºC). SDS resistant aggregates were detected using antibodies against Actin (B), HSP90-1 (C) or polyQ proteins (D).

E) SDS-PAGE of protein extracts from HEK293 cells were transfected with mRFP-Q74 and GFP-MC1 or mRFP-Q74 and GFP as a control. GFP antibody was used to detect GFP-MC1 and control GFP, while mCherry antibody was used to detect Q74 SDS-resistant aggregates. α-actin was used as a loading control.

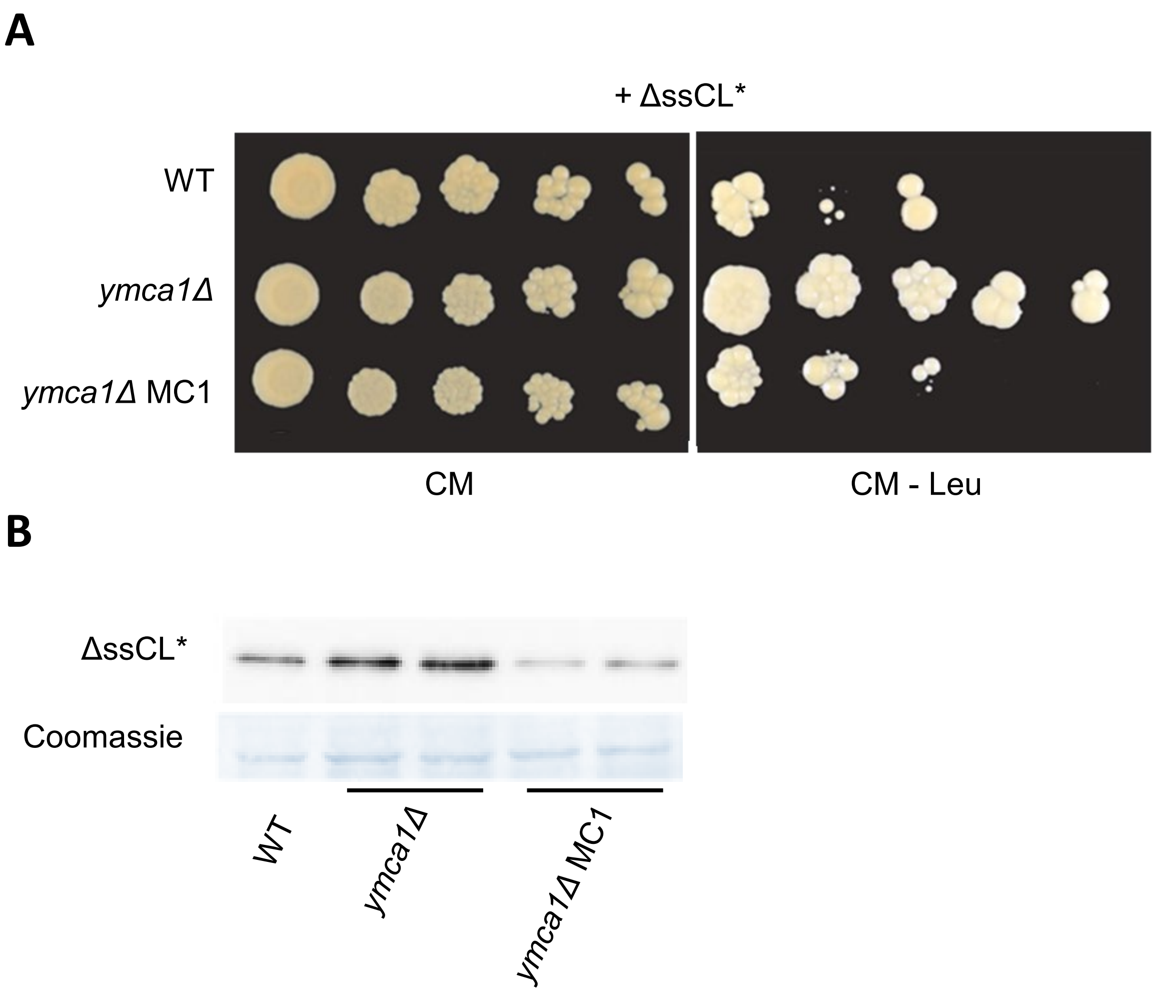

Figure S9. MC1 participates in the clearance of terminally misfolded proteins in yeast (Supports Figure 5).

A) Serial dilutions of wild type (WT), *ymca1Δ* mutant and *ymca1Δ* MC1-complemented cells expressing ΔssCL* were spotted on indicated media and incubated for 3 days at 30ºC. Enhanced growth on plates lacking leucine (CM – Leu) indicates stabilization of ΔssCL*, whereas reduced growth indicates increased degradation. Three independent experiments were performed.

B) SDS-PAGE of ΔssCL* levels of the strains shown in A. α-myc was used to detect ΔssCL*.

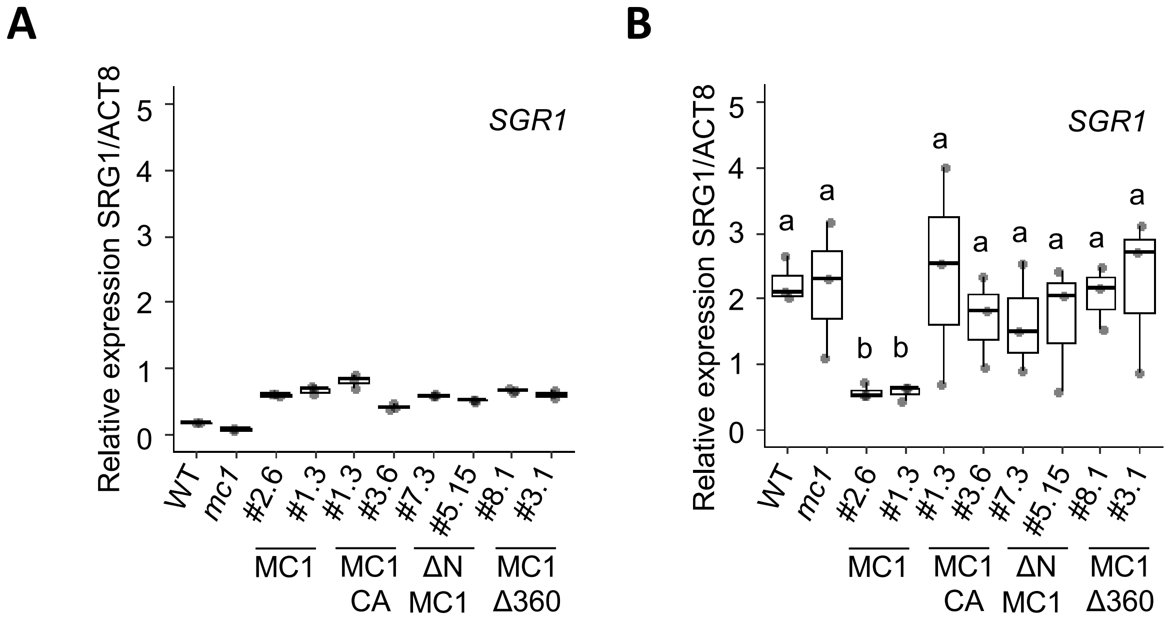

Figure S10. Relative expression of the senescence marker*SGR1 (AT4G22920)* (Supports Figure 6).

In A and B, qRT-PCR analysis of leaves of three-week-old Arabidopsis wild type, *mc1* mutants and *Pro35S:MC1-GFP mc1*, *ProMC1:MC1C220A-GFP mc1*, *ProMC1:ΔNMC1-GFP mc1* and *Pro35S:MC1Δ360loop-GFP mc1* grown for three weeks under controlled growth conditions (16 h light/8 h dark photoperiod) and covered for 5 days to induce senescence (B, Senescence) or left uncovered (A, Control). ACT8 was used as a reference. Three biological replicates are included. Means with different letters are significantly different at $P < 0.05$ (T-Test against WT).

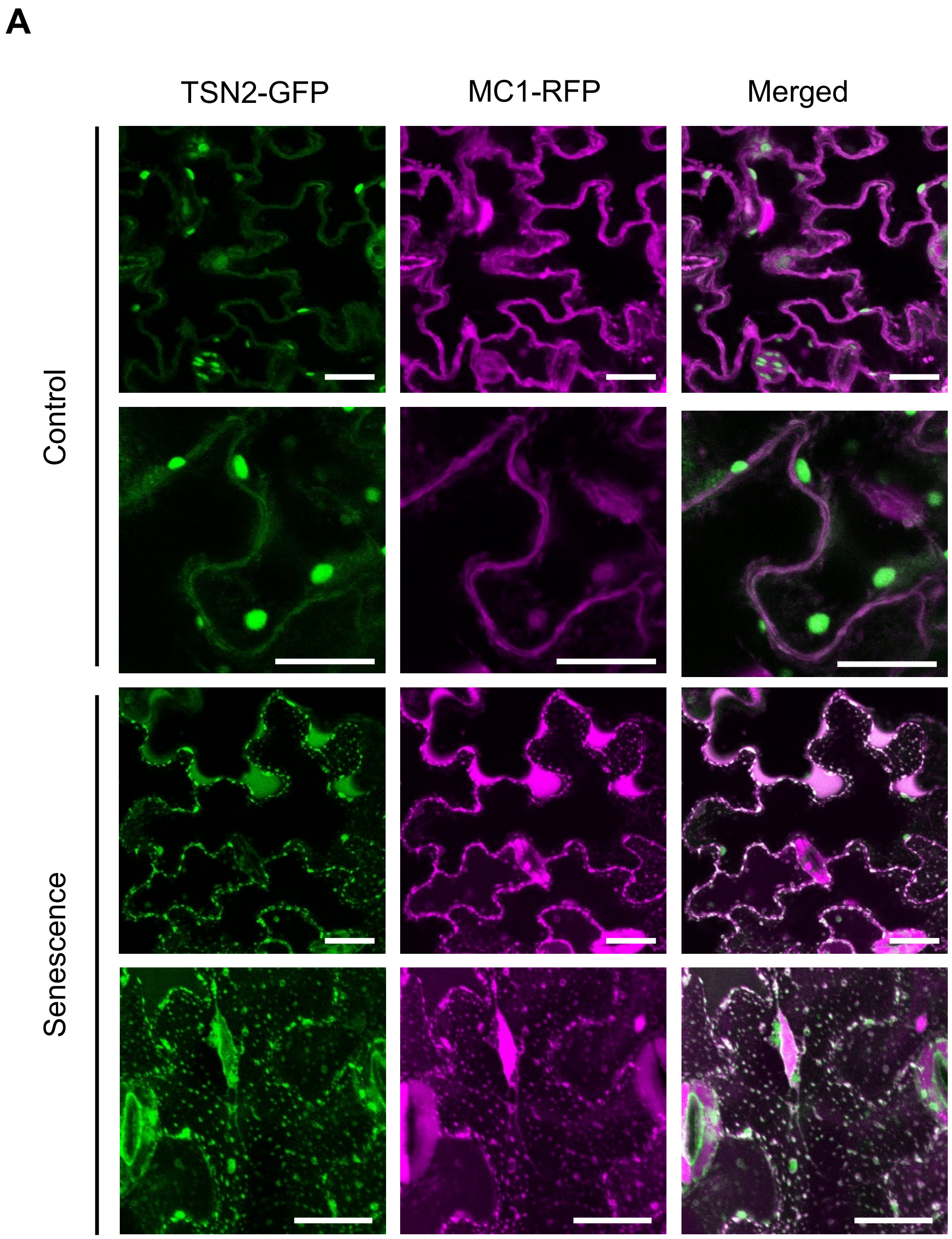

Figure S11. Dark-induced senescence results in the formation of TSN2 and MC1-containing cytoplasmic condensates (Supports Figure 6).

Representative confocal microscopy images of leaves of Arabidopsis *ProTSN2:TSN2-GFP* and *Pro35S:MC1-RFP* 3-week-old plants grown for three weeks under controlled growth conditions (Control, 16h light/8h dark photoperiod) and covered for 4 days to induce senescence (Senescence). Z-stacks with 5 slides (1 µm width) were performed. Bars = 20 µm.
