# Supplementary material for: Arabidopsis metacaspase MC1 localizes in stress granules, clears protein aggregates, and delays senescence
Source: Plant Cell. 2023 Jul 1;35(9):3325–44. doi: 10.1093/plcell/koad172 (PMC10473220; doi:10.1093/plcell/koad172)
Supplement: koad172_Supplementary_Data [file tpc22.01238.PeerReviewReport.pdf › tpc22.01238.PeerReviewReport.pdf]

Arabidopsis metacaspase MC1 localizes in stress granules, clears protein aggregates and delays senescence

Nerea Ruiz-Solaní[1], Jose Salguero-Linares[1], Laia Armengot[1,2], Jaime Santos[3], Irantzu Pallarès[3], Katarina P. van Midden[4], Ujjal J. Phukkan[1,¥], Seda Koyuncu[5], Júlia Borràs-Bisa[1], Liang Li[1], Crina Popa[1], Frederik Eisele[6], Anna Maria Eisele-Bürger[6], Sandra Malgrem Hill[6], Emilio Gutiérrez-Beltrán[7,8], Thomas Nyström[6], Marc Valls[1,2], Ernesto Llamas[9], David Vilchez[5,10,11], Marina Klemenčič[4], Salvador Ventura[3], Nuria S. Coll

Corresponding author: Nuria S. Coll (nuria.sanchez-coll@cragenomica.es)

Review timeline:

**TPC2022-RA-01238**	Submission received:	Dec. 19, 2023
	1st Decision:	Feb. 17, 2023 *revision requested*
**TPC2022-RA-01238R1**	1st Revision received:	April 18, 2023
	2nd Decision:	May 11, 2023 *accept with minor revision*
**TPC2022-RA-01238R2**	2nd Revision received:	June 7, 2023
	3rd Decision:	June 7, 2023 *acceptance pending, sent to science editor*
	Final acceptance:	June 13, 2023

REPORT: (The report shows the major requests for revision and author responses. Minor comments for revision and miscellaneous correspondence are not included. The original format may not be reflected in this compilation, but the reviewer comments and author responses are not edited, except to correct minor typographical or spelling errors that could be a source of ambiguity.)

TPC2022-RA-01238 1st Editorial decision – *revision requested* Feb. 17, 2023

We have received reviews of your manuscript entitled "Arabidopsis metacaspase MC1 localizes in stress granules, clears protein aggregates and delays senescence." Thank you for submitting your best work to The Plant Cell. The editorial board agrees that the work you describe is substantive, falls within the scope of the journal, and may become acceptable for publication, pending revision and potential re-review.

As you can see below, both reviewers found many strengths to your study. Reviewer #2 raises some concerns about the physiological links of your work. I encourage you to address this concern with data when feasible. When additional data is not feasible, I would be happy for additional language in the results and discussion that provide alternative explanations for your data.

-- Reviewer comments:

[Reviewer comments shown below along with author responses]

TPC2022-RA-01238R1 1st Revision received April 18, 2023

Reviewer comments and **author responses**:

Reviewer #1:

Manuscript entitled: Arabidopsis metacaspase MC1 localizes in stress granules, clears protein aggregates and delays senesence, describes discovery on one of metacaspases to be localizing into SG under stress conditions with the purpose of clearing the aggregates. Authors provided sufficient evidence that support the final conclusion. Manuscript is written in a good and clear way. All data support the conclusions.

I have few minor comments:

Line 27-28 - I suggest removing word "specifically" as it has been showed that similar SG-like structure was observed in plastids.

The word has been removed as indicated.

Figure 1. On the micrographs it looks that the puncta during 60 min of recovery are bigger, and they are more abundant, that is not reflected on the graph. Here there is a decrease in the number of SGs. In addition, on how many cells were measured? And how many times experiment was performed. n is not stated in the caption.

We have now included all the requested information regarding i) how many cells were measured, ii) how many times the experiments were performed and ii) the n for each experiment in Figure 1. We have also made sure that this information is present in all figure legends.

Regarding the reviewer's interesting observation that the puncta look bigger during time point 60 min of the recovery, we have performed new experiments to determine MC1-containing SG size dynamics, shown in Figures S2 and 4C. These experiments indicate that indeed, SGs grow over time until reaching a maximum around minute 80 of the recovery period and then start decreasing (Figure S2). Further, we show that deletion of the prodomain or the 360 loop in MC1 significantly alter the size of heat-induced SGs (Fig. 4C).

Another point, why TCTP was selected as SG marker? It has been shown recently to be in heat-induced SGs but there have been many others that we reported previously as key components of the core of SGs such TSN, RBP47. Authors included it as Supplemental but it would be good to have those SGs markers as key figure. Just a suggestion.

We agree with the reviewer that co-localization of MC1 with key core SGs markers would strengthen our data. Hence, we have generated Arabidopsis transgenic lines stably co-expressing *MC1-RFP* together with the core SG core marker *TSN2-GFP*. These plants have been used to perform co-localization and co- immunoprecipitation. Our data clearly show that MC1 co-localizes (new Fig. 1E, lower panel) and co- immunoprecipitates (new Fig. 1F, lower panel) with TSN2 upon heat stress. In addition, we have shown that dark-induced senescence also results in the formation of TSN2-MC1-containing puntcta (new Fig. S10).

Authors mentioned that MC1-3 are similar, authors should consider redundancy in the function claimed in the paper, unless there is clear indication that there is no redundancy.

Although at the molecular level there may be some redundancy in terms of molecular mechanism between MC1, MC2 and MC3 current evidence indicates that they might not participate in the same physiological processes based on:

i) Expression patterns of *MC1, MC2* and *MC3* are different (Coll et al., 2010; Pitsili et al., 2022). In particular, MC3 is specifically expressed in the phloem companion cells, which is very different from MC1 and MC2.

ii) Only the *mc1* mutant shows a clear proteostasis-related phenotype (Coll et al., 2014; Pitsili et al., 2022)

iii) Higher order *mc1-3* mutants result in a *mc1*-like phenotype (Salguero-Linares et al., manuscript in preparation).

References:

Coll NS, Vercammen D, Smidler A, Clover C, Van Breusegem F, Dangl JL, Epple P. Arabidopsis type I metacaspases control cell death. Science. 2010 Dec 3;330(6009):1393-7. doi: 10.1126/science.1194980.

Coll NS, Smidler A, Puigvert M, Popa C, Valls M, Dangl JL. The plant metacaspase AtMC1 in pathogen- triggered programmed cell death and aging: functional linkage with autophagy. Cell Death Differ. 2014 Sep;21(9):1399-408. doi: 10.1038/cdd.2014.50.

A phloem-localized Arabidopsis metacaspase (AtMC3) improves drought tolerance. Pitsili E, Rodriguez- Trevino R, Ruiz-Solani N, Demir F, Kastanaki E, Dambire C, de Pedro R, Vercammen D, Salguero-Linares J, Hall H, Mantz M, Schuler M, Tuominen H, Van Breusegem F, Valls M, Munné-Bosch S, Holdsworth MJ, Huesgen PF, Rodriguez-Villalon A, Coll NS. BioRxiv, doi: https://doi.org/10.1101/2022.11.09.515759.

Lines 176-178 Authors explained which constructs were use in the Figure 4 for localization studies. For consistency, compared plants should be harnessing the same constructs meaning the same promotor. For the mutated version of MC1, authors used native promoter.

Based on this comment we have realized that there was a mistake in the material and methods. All constructs used in Figure 4 all constructs contained the same 35S promotor.

Supplementary Figure 2 - It looks like Rbp47b and TSN localize in the plastids of protoplast?? Part of the foci colocalizes with MC1.. but signal is mostly distributed in the plastids, not like MC1 only cytosol.. is it the autofluorescence from the chlorophyll? If so, micrographs should be repeated to disconnect the RFP channel from chlorophyl.

The problem is that in our hands, using transient co-expression in protoplasts results in not very high levels of expression of the fluorescently tagged proteins. Therefore, we need to increase the laser intensity, which results in some level of autofluorescence, particularly in the chloroplast. However, co-localization of MC1 with either RBP47 or TSN2 in distinct cytoplasmic puncta is clear. In addition, we present complementary evidence of co-localization in

cytoplasmic puncta, using stable co-expression of MC1 with different markers in Arabidopsis transgenic lines (Fig. 1, Fig. S10). In our opinion, stable transgenics complement the protoplast data providing more robust evidence of co-localization.

Line 194-196 - As far as I know Rbp47b is a key component of the core of SGs. Authors mentioned that TSN is a is associated with the core that's why is more solid-like, while Rbp47b has big dynamics. Using this as reference, authors made assumption that MC1 as intermediate dynamic is perypherial /core protein. The belonging to the core or to the shell should not be defined by recovery here, as Rbp47b has high dynamic but is not a shell protein.

We have corrected the text describing our FRAP results as indicated.

What I would like to see as additional experiment is the gene expression level of senescence genes in experiments from Figure 6 A.

As requested, we have now included a quantitative PCR experiment (new Fig. S10) showing that a classical senescence marker gene (*SRG1*) (Ueda et al., 2020) is upregulated during the dark-induced senescence settings used in Figure 6A.

Reference: Ueda H, Ito T, Inoue R, Masuda Y, Nagashima Y, Kozuka T, Kusaba M. Genetic Interaction Among Phytochrome, Ethylene and Abscisic Acid Signaling During Dark-Induced Senescence in Arabidopsis thaliana. Front Plant Sci. 2020 May 21;11:564. doi: 10.3389/fpls.2020.00564.

<u>Reviewer #2:</u>

In this manuscript, the authors reach the following key and interesting findings:

1. MC1 is in stress granules
2. MC1 gets there through 2 IDRs that are present in it
3. C1 clears aggregates through its catalytic activity
4. MC1 has a role in senescence

Overall, I found the manuscript well-written and the figures simple and informative. At the same time, the link between aggregation and physiology is extremely weak. The biochemistry part is excellent and clear- I and raises no concerns to this end.

That MC1 gets into condensates upon heat stress is interesting, novel, and likely important. The identification of the 360 loop as the decisive factor for promotion into condensates is also interesting. Points 3 and 4 are less clear to me, and not well-supported by the current data provided.

I have some comments below which I hope will help the authors to build their story further. Major comments:

The link to senescence is not novel as the authors have published that MC1 links to senescence (see also line 88 of the current manuscript). Furthermore, is not followed to the extent that it should.

The reviewer is right in that we previously showed that *mc1* mutants show accelerated senescence (as stated in the introduction). Here, we show that overexpression of MC1 delays dark-induced senescence, which we did not observe before and we did not have the knowledge that we have now regarding the domains that regulate MC1 functions. To strengthen the link, we provide new data showing that dark- induced senescence results in the formation of puncta containing MC1 and the core SG marker TSN2 (new Fig. S11). In the future, we will certainly explore further the role of MC1 in senescence.

2. I do not doubt the localisation of MC1 in SGs, but how much of the total protein pool get there? is this significant? In yeast, for example, most proteins in condensates represent less than 20% of the total pool. Is this the clearance site? Mutants with reduced SGs: do they show symptoms of senescence?

The questions raised by the reviewer are extremely interesting, but unfortunately, tools to address them are not available in our field, to our knowledge. What has been shown so far is that mutants of SG core components show altered SG composition (Gutiérrez-Beltrán *et al.*, 2021). However, in plants no mutant has been identified that produces no SGs. This could be due to lethality associated to the absence of SGs in plants, although it has not been addressed in detail.

Reference: Gutierrez-Beltran E, Elander PH, Dalman K, Dayhoff GW 2nd, Moschou PN, Uversky VN, Crespo JL, Bozhkov PV. Tudor staphylococcal nuclease is a docking platform for stress granule components and is essential for

SnRK1 activation in Arabidopsis. EMBO J. 2021 Sep 1;40(17):e105043. doi: 10.15252/embj.2020105043.

3. Condensation is sensitive to concentration: levels of MC1Δ360-GFP vs WT variant?

We think that, as the reviewer suggests, condensation may be sensitive to concentration in our system. However, we do not have definitive evidence to show that. The MC1Δ360-GFP lines initially used showed similar protein levels to the Wt variant and formed barely any condensate upon heat stress. However, these lines were silenced quickly and later we have only been able identify new MC1Δ360-GFP lines were protein levels are higher than Wt (see new Figure S6), which are also able to form higher number of SGs (new Fig. 4B), although they are significantly smaller in size than Wt SGs (new Fig. 4C).

4. Line 196: makes no sense to me. How someone can tell this using FRAP?

We have corrected the paragraph in which we describe our FRAP results. Indeed, we cannot make such affirmations from a photobleaching experiment. Thus, we have focused our results in the dynamics of MC1 and its mutants variants (new Fig. 4D,E).

5. Dark-induced senescence is not the same as age-dependent senescence. Authors here should try harder to disentangle the connection between SGs and senescence, for example, for example providing dynamics during a physiological senescence process. Darkness has been shown to include condensates with properties of mRNA storage, and thus authors might be looking at something completely different.

We agree that dark-induced senescence is not exactly the same as age-dependent senescence, although the first is commonly used as a proxy for the second and this has been generally accepted by the scientific community and well documented. When trying to use assays to monitor different parameters of age- induced senescence, we found extremely high variability. We followed published protocols such as Minina et al., 2013 and even contacted the authors, which informed us that the results are very variable depending on the repetition and conditions. Therefore, we opted for dark-induced senescence for robustness and consistency. We have added a new figure showing upregulation of a classical senescence marker in our dark-induced senescence settings (new Fig. S10). Still, we plan to continue working to optimize the conditions to study MC1 during age-induced senescence, which beyond the scope of the present work.

Reference: Minina EA, Sanchez-Vera V, Moschou PN, Suarez MF, Sundberg E, Weih M, Bozhkov PV. Aging Cell. 2013 Apr;12(2):327-9. doi: 10.1111/acel.12048.

1. The story would be strengthened by trials in vitro to carefully check the links between concentration and LLPS of the different MC1 variants.

This is certainly interesting but for now we can only work in vitro with a single variant that is MC1 devoid of the two main IDRs: the prodomain and the 360 loop, as shown in Fig. 3 and Fig. S4. We are currently doing single amino acid deletions of all Type I metacaspases to determine their solubility/LLPS properties in vitro and in vivo and hopefully we will have more information in that regard in the near future.

**TPC2022-RA-01238R1 2nd Editorial decision – *accept with minor revision***	**May 11, 2023**

We have received reviews of your manuscript entitled "Arabidopsis metacaspase MC1 localizes in stress granules, clears protein aggregates and delays senescence." On the basis of the advice received, the board of reviewing editors would like to accept your manuscript for publication in The Plant Cell. This acceptance is contingent on revision based on the comments of our reviewers. In particular, please consider the following:

Reviewer #2 requests that you more fully discuss the caveats from their comments #2 and #5 from their original review:
2. I do not doubt the localisation of MC1 in SGs, but how much of the total protein pool get there? is this significant? In yeast, for example, most proteins in condensates represent less than 20% of the total pool. Is this the clearance site? Mutants with reduced SGs: do they show symptoms of senescence?
5. Dark-induced senescence is not the same as age-dependent senescence. Authors here should try harder to disentangle the connection between SGs and senescence, for example, for example providing dynamics during a physiological senescence process. Darkness has been shown to include condensates with properties of mRNA storage, and thus authors might be looking at something completely different.

In particular, they would like you to more fully explore how your data relates to the data from the publications listed below and also consider the localization of MC1to PBs, which regulates darkness-related phenotypes.

Minina EA, Sanchez-Vera V, Moschou PN, Suarez MF, Sundberg E, Weih M, Bozhkov PV. Aging Cell. 2013 Apr;12(2):327-9. doi: 10.1111/acel.12048.

Gutierrez-Beltran E, Elander PH, Dalman K, Dayhoff GW 2nd, Moschou PN, Uversky VN, Crespo JL, Bozhkov PV. Tudor staphylococcal nuclease is a docking platform for stress granule components and is essential for SnRK1 activation in Arabidopsis. EMBO J. 2021 Sep 1;40(17):e105043. doi: 10.15252/embj.2020105043.

--- Reviewer comments:

Reviewer #1 (Comments for the Author):

Authors critically addressed all the comments from my side. Manuscript and figures are even more clear. I am satisfied with the revision therefore I can suggest acceptance of the manuscript.

Reviewer #2 (Comments for the Author):

The authors have responded to some of my comments but still, I am not entirely convinced that the link to SGs-MC1 drives the observed phenotypes. The authors could have done more to address points 2 and 5, as I feel these are important for the work. I suggest either the authors discuss these caveats thoroughly or perform additional experiments to strengthen the links between the observed phenotypes.

TPC2022-RA-01238R2 2nd Revision received	June 7, 2023

Reviewer comments and **author responses**:

2. I do not doubt the localisation of MC1 in SGs, but how much of the total protein pool get there? is this significant? In yeast, for example, most proteins in condensates represent less than 20% of the total pool. Is this the clearance site? Mutants with reduced SGs: do they show symptoms of senescence?

> **We feel that we already answered in the previous round of responses to reviewers, but this information was not included in the manuscript, which now is, as follows:**
>
> **Line 343: How much of the total MC1 protein pool localizes to SGs remains to be established.**
>
> **Line 392: Still, it remains to be defined whether MC1-containing condensates are the physiological sites of protein aggregate clearance and what is the exact MC1 mode of action therein.**
>
> **Line 442: In fact, plants lacking SG core components show altered SG composition (Gutiérrez-Beltrán *et al.*, 2021), but so far mutants completely devoid of SGs have not been identified. This could be due to lethality associated to the absence of SGs in plants, although it has not been addressed in detail.**

5. Dark-induced senescence is not the same as age-dependent senescence. Authors here should try harder to disentangle the connection between SGs and senescence, for example, for example providing dynamics during a physiological senescence process. Darkness has been shown to include condensates with properties of mRNA storage, and thus authors might be looking at something completely different.

> **Line 416: Considering that in our system we use dark as a trigger for senescence, one may speculate that the observed condensates are processing bodies (PBs) rather than SGs, since PBs have been shown to act as mRNA reservoirs under dark conditions (Jang *et al.*, 2019). In contrast to SGs, PBs are constitutively present in the cell, but they have been shown to increase in number and size during stress conditions and exchange proteins and RNAs with SGs (Chicois et al., 2018; Gutierrez-Beltrán et al., 2015; Solis-Miranda *et al.*, 2023), therefore a functional link may exist between them. A recent metaproteomic analysis of stress-induced condensates showing distinct protein signatures for SGs and PBs has revealed that MC1 is only present in SGs across various stress conditions (Solis-Miranda *et al.*, 2023). Still, it will be interesting to determine the composition of MC1-containing condensates under natural and dark-induced senescence to define the degree of resemblance to stress-induced condensates.**

TPC2022-RA-01238R2 3rd Editorial decision – *acceptance pending*	June 7, 2023

We are pleased to inform you that your paper entitled "Arabidopsis metacaspase MC1 localizes in stress granules, clears protein aggregates and delays senescence" has been accepted for publication in The Plant Cell, pending a final editorial review by a science editor.

Final acceptance from Science Editor **June 13, 2023**
